# Air-conditioning replacement to enhance the reliability of renewable power systems under extreme weather risks

**DOI:** 10.1093/pnasnexus/pgaf230

**Published:** 2025-07-23

**Authors:** Lei Zhu, Zhuang Liang, Zhihao Yan, Xi Ming, Hongbo Duan, Bin Su, Shouyang Wang

**Affiliations:** School of Economics and Management, Beihang University, No. 37 Xueyuan Road, Haidian District, Beijing 100191, China; School of Economics and Management, Beihang University, No. 37 Xueyuan Road, Haidian District, Beijing 100191, China; School of Economics and Management, Beihang University, No. 37 Xueyuan Road, Haidian District, Beijing 100191, China; School of Economics and Management, University of Chinese Academy of Sciences, No. 19A Yuquan Road, Haidian District, Beijing 100190, China; School of Economics and Management, University of Chinese Academy of Sciences, No. 19A Yuquan Road, Haidian District, Beijing 100190, China; Energy Studies Institute, National University of Singapore, 29 Heng Mui Keng Terrace, Singapore 119620, Singapore; Department of Industrial Systems Engineering and Management, National University of Singapore, 1 Engineering Drive 2, Singapore 117576, Singapore; School of Economics and Management, University of Chinese Academy of Sciences, No. 19A Yuquan Road, Haidian District, Beijing 100190, China; Academy of Mathematics and Systems Science, Chinese Academy of Sciences, No. 55 Zhongguancun East Road, Haidian District, Beijing 100190, China

**Keywords:** air-conditioning, extreme weather risk, power system, reliability, economic and environmental benefits

## Abstract

The increasing demand for residential heating and cooling significantly affects power systems, especially during extreme weather events. The replacement of outdated room air-conditioning (RAC) with a high-efficiency model demonstrated considerable potential in alleviating this effect. In this study, the impacts of extreme warm, cold, and drought events on power demand and supply are explored. By simulating residential heating and cooling loads in southern Chinese cities and integrating these loads into a provincial power dispatch model, we confirm the positive role of RAC replacement in carbon mitigation and power system cost reductions. It also enhances the power system reliability, especially facing extreme weathers. Specifically, RAC replacement may reduce peak power demand by up to 12.2% for advanced high-efficiency units. Compared with the battery storage system, RAC replacement has better cost and emission reductions, given the same budget. This research highlights the pivotal role of improving residential energy efficiency in the transition toward a green power system in China, especially the pronounced benefits of RAC replacement in enhancing energy resilience when facing extreme weather risks.

Significance Statement Extreme weather events strain power grids, a challenge compounded by the transition to variable renewables like wind and solar. This study demonstrates that replacing inefficient room air-conditioners (RACs) with high-efficiency units is a powerful demand-side solution. Simulating the power system of southern China, we find that this strategy significantly reduces peak electricity demand (by up to 13.5%), carbon emissions, and system costs. Critically, it enhances grid reliability, lowering the risk of load losses during extreme weather events. By comparing this approach with adding battery storage under the same budget, the research proves RAC replacement as a vital, cost-effective pathway toward stable, low-carbon energy systems.

## Introduction

The United Nations Climate Change Conference (COP28) has proposed pledges to enhance energy efficiency and diminish cooling-related emissions, aiming to cap global warming at 1.5 °C (1, 2). China, which has experienced the sharpest increase in cooling demand in the 21st century, has improved the energy efficiency standards of room air-conditioning (RAC) systems, increasing the energy efficiency ratio—which indicates the cooling capacity generated per watt of electricity consumed—from 2.5 to 5.8 (3–6). Nevertheless, the long lifespan of RAC systems and the steep initial costs of energy-efficient models often deter residents from upgrading their units (7–9). Assessing the potential of RAC replacement is critical for offsetting the escalating challenge of global warming. In southern China, the market for space heating and cooling is particularly active, with average RAC ownership per household reaching 1.69 units in 2020; this value is more than double that of households in northern regions (10–12). The lack of centralized heating and increasing summer warming make the situation more severe (13–16), which, in turn, challenges the power system during peak load times, particularly under extreme weather conditions (17–20).

With the intensification of global warming, extreme weather events are increasing in frequency (21–24). As China has experienced numerous extreme weather events, including record-breaking high and low temperatures in 2023, there is an increasing acknowledgment of the vulnerability of renewable energy systems to weather variations (25–29). Moreover, the commitment of China to reach peak carbon emissions by 2030 and achieve carbon neutrality by 2060 necessitates the increase in renewable energy use and reduction in dependency on coal-fired power (30, 31). Extreme weather events can fluctuate power demand and supply, posing significant challenges to achieving carbon neutrality while maintaining power system stability. Therefore, solely focusing on fluctuations in load or renewable generation is inadequate for addressing the stability challenges of climate and power systems (32–35).

Recent research has made notable progress in exploring the interplay between climate change, electricity demand, and power system reliability. Amonkar et al. (36) examined how climate change influences peak heating and cooling demands in the United States, highlighting the challenges posed by increased RAC adoption in regions experiencing rapid temperature rises, and the impact of weather-induced peak demand fluctuations on the Texas power system has also been studied, emphasizing the need for better winter preparedness (37). Farghali et al. (38) and Xu et al. (29) explored pathways for reducing energy consumption and decarbonizing the electricity sector but did not address how these strategies interact with climate change. Akdemir et al. (39) highlighted the role of cooperative transmission planning in enhancing system reliability during extreme weather, while Duan et al. (10) examined how climate change is driving increased air-conditioner sales and electricity consumption in China.

Building on these foundational studies, our research focuses on an underexplored yet crucial dimension: replacing outdated RACs with high-efficiency models as a demand-side strategy to address the challenges of peak load, extreme weather, and decarbonization. By integrating residential heating and cooling loads into a provincial power dispatch model, we assess the impact of RAC replacement on power system costs, emissions, and reliability, particularly under extreme weather in southern China. This study extends the literature by demonstrating the superior cost-effectiveness and emission reduction potential of RAC replacement compared with other measures such as battery storage systems.

**Fig. 1.**
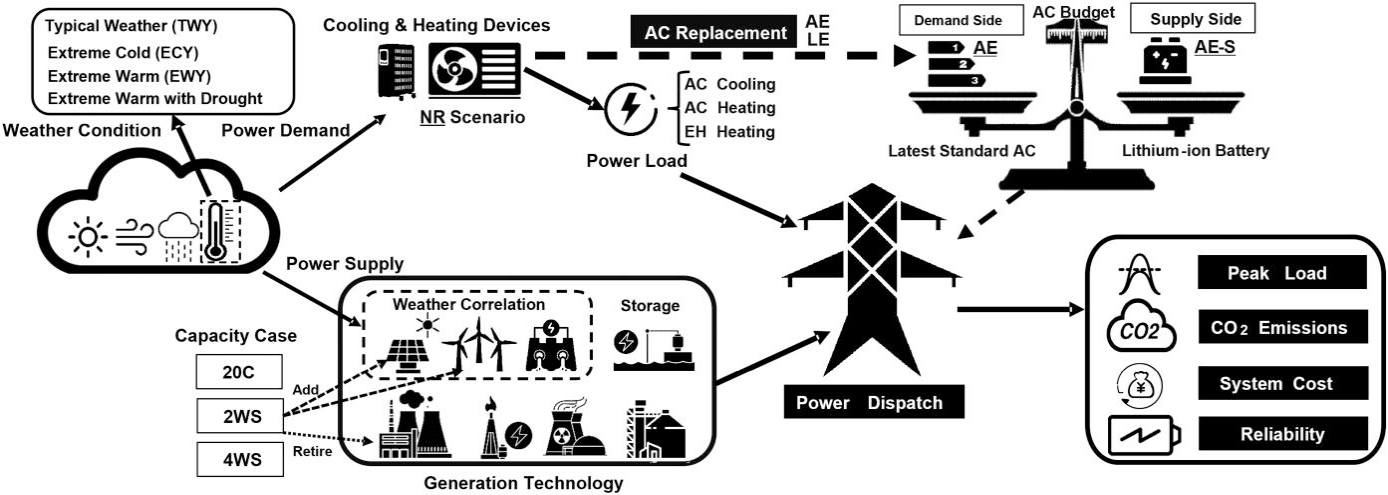
Understanding the influences of extreme weather and RAC replacement for the power system.

In our research, we employ a bottom-up approach to gauge the energy efficiency of existing RAC systems in southern Chinese cities and incorporate these loads into a provincial power dispatch model to assess the impact of RAC replacement on the power system (Fig. 1). We aim to provide a comprehensive understanding of the role of RAC replacement in ensuring power system stability and in facilitating the transition toward carbon neutrality. To depict the impact of weather factors, it is not only considered in calculating heating and cooling loads of RAC replacement together with other factors, such as urban income and occupancy, but also been accounted in the power transmission and renewable energy generation.

To verify the effectiveness of RAC replacement in different scenarios, we implement various weather and power capacity conditions (Tables 1 and S1). Three weather scenarios are constructed based on different temperature conditions—a typical weather year (TWY), an extreme cold year (ECY), and an extreme warm year (EWY)—supplemented by an additional scenario: extreme warm and drought. These scenarios encompass ambient temperature, precipitation, wind speed, and solar radiation as key weather conditions. We assume that no RAC replacement occurred between 2000 and 2021 (NR). In addition, we explore scenarios involving the replacement of these devices with systems featuring two different efficiency levels: lowest energy efficiency (LE) and advanced energy efficiency (AE) (40). To validate the impact of RAC replacement, we introduce a battery storage scenario with an AE budget (AE–S) (37). The power system simulation incorporates seven generation technologies (coal, gas, biomass, nuclear, hydro, wind, and solar) and one storage technology (pumped storage) (41). Further, we define three capacity scenarios to assess the impact of RAC replacement with power system low-carbon transition: 20C, maintaining 2020 generation capacities; 2WS, doubling wind and solar capacities relative to 2020 levels; and 4WS, quadrupling wind and solar capacities relative to 2020 levels.

**Table 1.** List of scenarios.

RAC replacement status	Description
NR	No replacement before 2021
LE	Replacement of pre-2009 RACs with lowest energy efficiency level
AE	Replacement of pre-2009 RACs with advanced energy efficiency level
AE-S^a^	Adding battery storage with the AE budget and no RAC replacement
Weather conditions^b^	
TWY	Weather conditions with average temperature
ECY	Weather conditions with the lowest temperature
EWY	Weather conditions with the highest temperature
Drought^c^	Weather conditions with the highest temperature and drought
Capacity cases	
20C	2020 electric power mix
2WS	Power mix with wind and solar capacities doubled relative to 2020, paired with the retirement of coal-fired units
4WS	Power mix with wind and solar capacities quadruple relative to 2020, paired with the retirement of coal-fired units

^a^AE–S is the additional scenario of AE to verify the validity of RAC replacement compared with a 4-h lithium-ion battery storage.

^b^Weather conditions are determined based on hourly temperature data from various years spanning 1993 to 2022, with each hour selected from the same time across different years.

^c^The drought scenario is included as an extension of EWY, with hydropower output reduced and other conditions unchanged.

We find that RAC replacement substantially benefits the power system, especially when addressing extreme weather events and transitioning to a low carbon level. Extreme weather events increase the heating and cooling load, and RAC replacement significantly reduces these loads, particularly during peak demand. Moreover, RAC replacement notably decreases carbon dioxide (CO_2_) emissions, system costs, and the risk of load losses within power systems. Notably, extreme cold weather poses the greatest challenge to power systems, with increased electricity demand and reduced renewable energy generation. Therefore, the potential advantages of RAC replacement should be highlighted as China attempts to reach its carbon peak and carbon neutrality targets, particularly considering the growing frequency of extreme weather events.

## Results

### Peak load reduction through RAC replacement under extreme weather risk

**Fig. 2.**
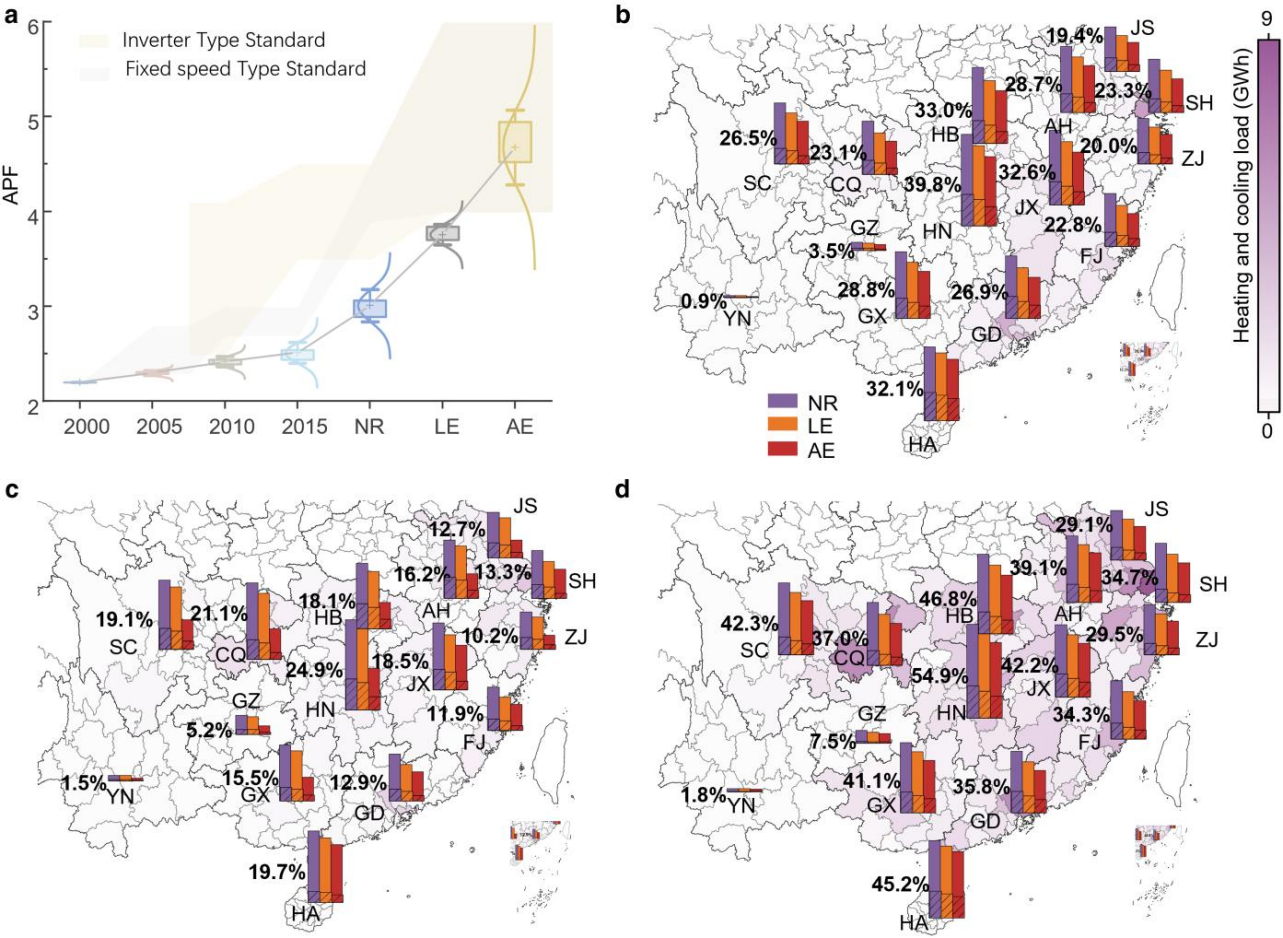
The influence of heating and cooling load on the power system. a) WAPF change of all existing air-conditionings among cities. The box chart shows the WAPF changes over time and replacement scenarios, and the line connecting the different scenarios shows the change in the average WAPF of all cities. The curve shows the normal distribution of WAPF. The upper and lower limits of the shadow represent the lowest and most advanced APF standards for each type of RACs sold in that year. b–d) The heating and cooling load and its proportion under peak load hour in NR scenario with 20C case under TWY (b), ECY (c), and EWY (d) conditions, respectively. The hour that records the highest percentage of heating and cooling demand in power load is identified as the peak load hour. The map shows city-level heating and cooling load at the peak hour. The bar chart filled with solid colors and diagonal lines shows the proportion of peak and average heating and cooling load in provincial power load, respectively. The chart contains data from Anhui (AH), Chongqing (CQ), Fujian (FJ), Guangdong (GD), Guangxi (GX), Guizhou (GZ), Hainan (HA), Hubei (HB), Hunan (HN), Jiangsu (JS), Jiangxi (JX), Shanghai (SH), Sichuan (SC), Yunnan (YN), and Zhejiang (ZJ). The bar color distinguishes the different RAC replacement scenarios (see Tables S3 and S4 for detailed setting).

We calculate the city-level energy efficiency and ownership status of existing RAC systems between 2000 and 2021 and simulate the hourly heating and cooling loads for urban residents under different weather scenarios and RAC replacement status (Fig. 2). The resident heating and cooling models are presented in the “Materials and methods” section and Note S1. The peak load is defined as the total load in the hour with the highest proportion of heating and cooling load. There are significant differences in the peak load during extreme weather events across the provinces, and RAC replacement consistently decreases the peak load. This effect is more pronounced during extreme weather events than during TWYs.

We use annual performance factor (APF) to calculate a weighted APF (WAPF) based on RAC sales across different years, representing the average efficiency level of all existing RACs (Note S1), in which APF is an indicator that reflecting energy efficiency over a year by considering seasonal temperature variations. The average WAPF for all cities increased from 2.2 in 2000 to 3.0 in the NR scenario (Fig. 2a). RAC replacements are projected to further enhance the average WAPF to 3.76 in the LE scenario and 4.67 in the AE scenario, reducing peak loads by up to 5.6 and 10.2%, respectively.

Most provinces, except for Guizhou and Yunnan, face large peak load pressure under TWY conditions, and RAC replacement might ease their pressures. The peak load ratio is considerably greater than the average load, exhibiting a regional pattern with fluctuations ranging between 0.9 and 39.8% (Fig. 2b). In contrast, the average load ratio varies slightly from 0.1 to 6.5%, with peak loads being 5.4 to 9.9 times greater than the average. Inland provinces (except Guizhou and Yunnan) benefit more from peak load ratio reduction by RAC replacement since they consistently exhibit higher peak and average load ratios than coastal provinces. (The classification of provinces, including whether they are coastal or inland, as well as their regional power grids and climate zones, is provided in the Table S2.) In the LE scenario, Anhui, Chongqing, Hubei, Hunan, and Jiangxi achieve at least a 4.3% reduction in peak load ratio, which increases to 8.2% in the AE scenario. The situation changes for Guizhou and Yunnan, in which the peak loads are not considerably reduced, only 0.9 and 0.5%, respectively in the AE scenario. This phenomenon can largely be ascribed to the relatively mild climatic conditions that are prevalent in these regions, which consequently reduce the rate of RAC ownership. The data from 2021 reflect this trend, showing household RAC ownership rates in Yunnan and Guizhou of only 0.48 and 0.11 units per household, respectively.

The peak load ratio variations under ECY conditions span between 1.5 and 24.9% of the provinces in the NR scenario. Although the load pressure under ECYs is lower compared with TWYs, RAC replacement shows greater load reduction potential (Fig. 2c). While facing extreme cold and warm weather events, the peak load and average load decrease in ECYs and increase in EWYs in most provinces (Fig. 2c and d). Specially, Guizhou and Yunnan increased their peak and average loads in ECYs compared with TWYs, and their mild climate caused low cooling or heating demand in TWYs, but the low temperature in ECYs increased their peak loads. Since cold weather increases the heating demand, under ECY conditions, RAC replacement significantly reduces loads in the hot summer and cold winter (HSCW) zone, which includes the provinces of Anhui, Chongqing, Hubei, Hunan, Jiangsu, Jiangxi, Shanghai, Sichuan, and Zhejiang, particularly in the AE scenario. Because of modifications in heating appliances within the AE scenario, the peak load reduction is notably greater than that in the LE scenario. The provinces in the HSCW zone achieve extensive reductions, averaging 9.2%, compared with 5.6% in the hot summer and warm winter (HSWW) zone, which contains the provinces of Fujian, Guangdong, Guangxi, and Hainan. Compared with the NR scenario, the highest peak load ratio reductions for the LE and AE scenario appear in Jiangxi and Hunan, reaching 3.3 and 13.5%, respectively.

Extreme warm weather events impose substantial load pressure to power systems. Compared with TWY conditions, all provinces experience increases in both peak load and average load under EWY conditions (Fig. 2d). Hunan Province has the highest peak load ratio among all provinces, reaching 54.9% in the NR scenario. However, the highest load reduction is observed in Chongqing, which achieves a 12.4% reduction in the AE scenario. This notable reduction in load is largely due to Chongqing's high ownership of outdated RAC. In 2009, the household RAC ownership in Chongqing was 1.51 units per household, significantly higher than Hunan's 1.03 units per household. Inland provinces (except Guizhou and Yunnan) also exhibit a higher peak load proportion and greater reduction in under EWY conditions, reaching an average peak load ratio of 43.7%, exceeding the 35.7% level observed in coastal provinces. Notably, Chongqing, Hubei, Hunan, and Sichuan all experience more than 13% peak load ratio increases compared with TWYs, significantly increasing the pressure on the central China grid (e.g. Chongqing, Henan, Hubei, Hunan, Jiangxi, and Sichuan).

### Disparity of CO_2_ emissions with load variations

**Fig. 3.**
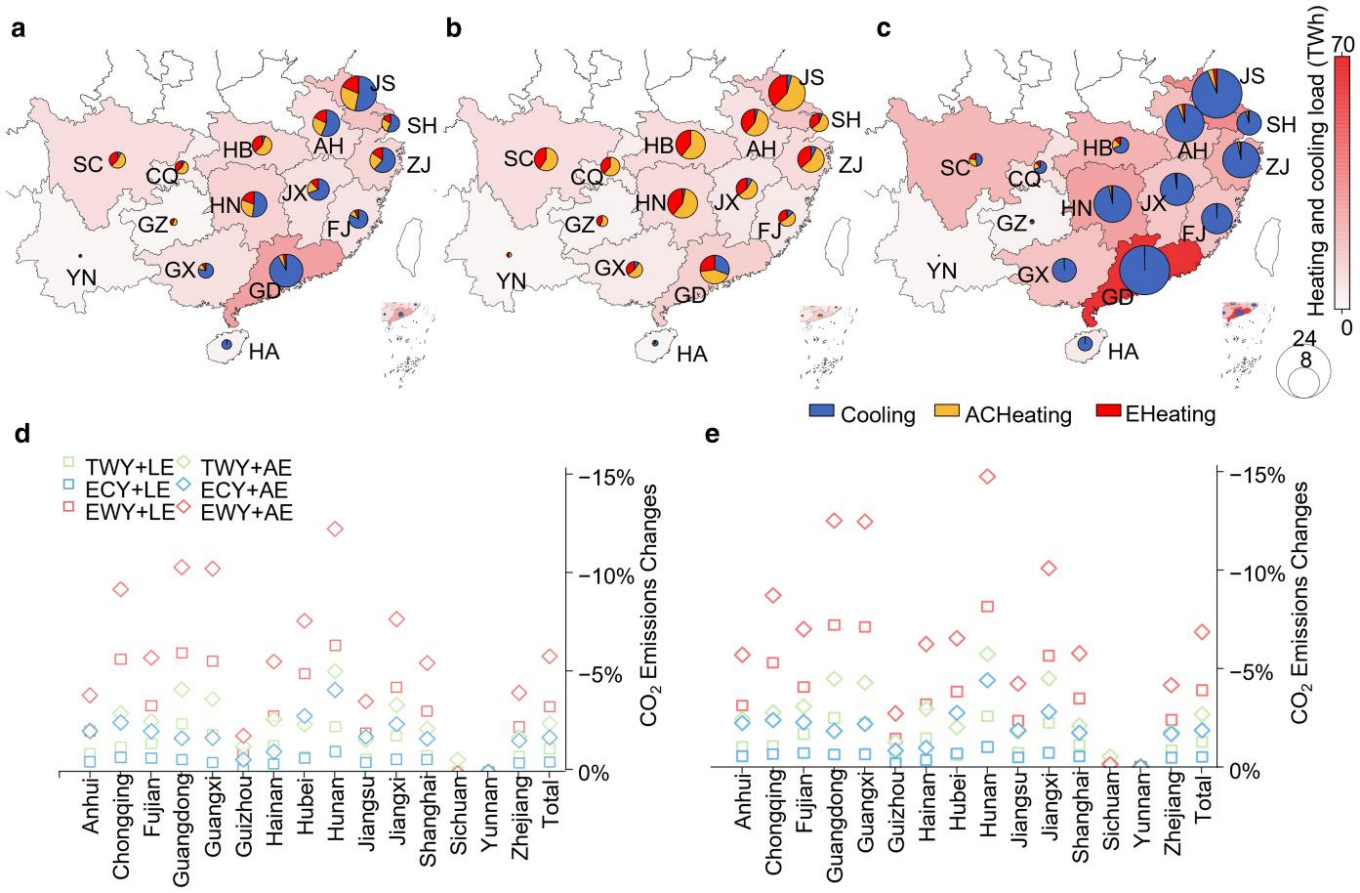
Changes in CO_2_ emissions from heating and cooling under different weather scenarios. a–c) The annual heating and cooling load and its CO_2_ emissions from different sources under the NR scenario with the 20C case in TWY (a), ECY (b), and EWY (c) conditions, respectively. To include the impact of power transmission on CO_2_ emissions, we calculate the CO_2_ emissions from electricity consumption side. The map illustrates the annual provincial heating and cooling load distribution, while the bar chart delineates the emissions levels, with distinct colors indicating emissions from cooling, heating by RACs (ACHeating), and heating by electric heaters (EHeating). d and e) The annual provincial CO_2_ emissions reductions from total power load under different combinations of weather conditions and RAC replacement scenarios in the 20C case (d) and 2WS case (e). The “Total” shows the sum of all research provinces' CO_2_ emission reductions. The CO_2_ emissions in the NR scenario under the same weather condition and capacity case are set as baseline.

We integrated hourly heating and cooling loads into an interregional power dispatch model and considered the impact of weather on renewable energy generation (hydropower, wind energy, and solar energy) to calculate CO_2_ emissions across various provinces in combinations of weather scenarios. In addition to RACs, distributed electric heaters, such as fan heaters, electric blankets, and infrared heaters, are widely used in southern China. To accurately estimate the heating electricity consumption of RACs, we separated the heating load into air-conditioning heating (ACHeating) and electric heater heating (EHeating) (Note S1). We find that while cooling contributes more to load increases than heating, the increase in CO_2_ emissions from cooling is considerably lower than that from heating (Fig. 3). This discrepancy is attributed to the hindered increase in renewable generation at elevated temperatures. RAC replacement benefits from increased renewable energy utilization, resulting in significant CO_2_ emission reduction, particularly under TWY and EWY conditions.

Diverse generation technologies and regional weather patterns result in asynchronous changes in CO_2_ emissions with load variation. We illustrate the CO_2_ emissions resulting from heating and cooling and from changes in the NR scenario in the 20C case across different weather scenarios in Fig. 3a–c. During TWYs, the proportion of cooling emissions increases from north to south and from inland to coastal provinces (Fig. 3a). Cooling contributes more than 88.8% of the heating and cooling emissions in the HSWW zone, while the proportion of emissions varies from 55.3 to 69.4% in the HSCW zone due to the large-scale hydropower capacities in Hubei and Sichuan, which support the cooling demand of these provinces with substantial hydropower generation on warm days. Under ECY conditions, most provinces experience a reduction in heating and cooling loads while having an increase in CO_2_ emission levels (Fig. 3b). In cold weather, all provinces reduce their cooling load and increase their heating load. However, cold weather creates unfavorable conditions for renewable generation, which increases CO_2_ emissions.

In contrast to ECYs, during EWYs, most provinces experience an increase in heating and cooling loads and a relatively low increase in CO_2_ emissions (Fig. 3c). Warm weather leads to favorable solar radiation and precipitation, boosting renewable generation and decreasing the CO_2_ intensity (Fig. S4). As a result, the increase in emissions across provinces is less than the rise in load. However, while EWYs capture the effects of global warming on precipitation (42–44), EWYs may not fully account for the potential of extreme drought triggered by high temperatures. To address this, we also present the results under a scenario combining extreme warm weather with drought conditions, as discussed in the section “Positive Role of RAC Replacement in Tackling Drought Weather.” In EWYs, all provinces experienced an average load increase of 132.2%, while emissions increased by 96.6%. Despite this considerable load surge, provinces such as Chongqing, Guizhou, Hubei, Sichuan, and Yunnan—benefiting from hydropower capabilities—experience a reduction in CO_2_ emissions.

The impact of RAC replacement on reducing CO_2_ emissions becomes noteworthy under EWYs (Fig. 3d). Under the 20C case, overall, RAC replacement can decrease the CO_2_ emissions by 1.2 and 2.5% under the TWY conditions with the LE and AE cases, respectively. EWYs bring both high cooling loads and increased solar power generation to the power system. The load reduction from RAC replacement makes it easier for the power system to meet cooling demands with enough renewable capacity, resulting in significant carbon reduction potential. These reductions experienced in the LE and AE scenarios notably increase to 3.3 and 5.9%, respectively, under EWY conditions and decrease to 0.5 and 1.7%, respectively, under ECY conditions.

Under the 2WS case, the reduction in CO_2_ emissions is greater than that under the 20C case across all weather conditions. In EWYs, total CO_2_ emissions reduce by 3.9 and 6.9% in the LE and AE scenarios, respectively. Provinces in the HSWW zone and Central China power grid, except for Sichuan, which face significant peak load challenges, exhibit increasingly pronounced CO_2_ emissions reduction effects through RAC replacement. The higher CO_2_ reduction is also observed during TWYs and ECYs. Specifically, the total reduction in TWYs and ECYs exceeds that of the 20C case by 0.2 and 0.1%, respectively, when the same AE scenario is applied. Sichuan and Yunnan have the lowest emissions and costs per kilowatt-hour (Fig. S3). As the loads in the provinces decrease, those with higher generation costs can meet most of their needs by purchasing low-cost electricity from other provinces such as Sichuan. Consequently, these provinces' emission reduction effect is partially transferred to these other provinces, making its own emission reduction effect less pronounced. In the 4WS case, the trends are similar across weather conditions, but the reductions in CO_2_ emissions are even higher (Fig. S5a).

### Cost-effectiveness and enhanced power system reliability

**Fig. 4.**
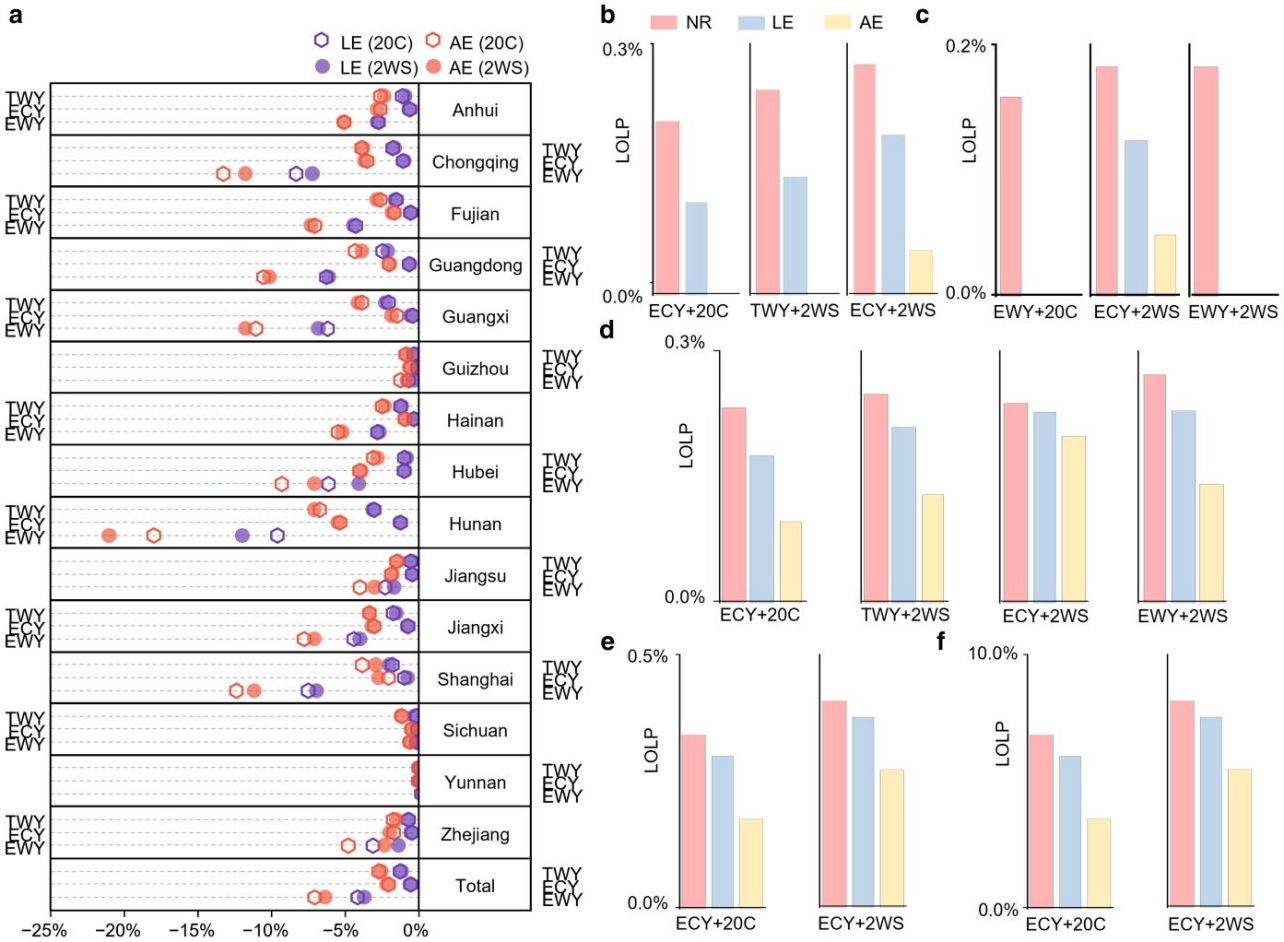
Impact of RAC replacement on system cost and LOLP. The figure shows the annual provincial system cost (a) and LOLP (b–f) changes from total power load under different replacement scenarios, capacity cases, and weather conditions. a) Each province has three rows of scatterplots, representing TWY, ECY, and EWY conditions, respectively. The changes in cost are computed by comparing values with the NR scenario under the same weather condition and the same capacity case. b–f) The changes in LOLP in Chongqing (b), Hunan (c), Hubei (d), Guangxi (e), and Sichuan (f), corresponding to various replacement scenarios. Each subfigure consists of three bar charts representing the weather scenarios and capacity case with LOLP. To mitigate the influence of power load reduction due to RAC replacement in LOLP calculation, we utilized the total power load from the NR scenario as the denominator for LOLP in both LE and AE scenarios.

Moreover, we estimate changes in system cost, including fuel, startup, shutdown, operation and maintenance costs, in addition to the stability of the power system. Our analysis reveals a substantial cost-saving effect resulting from RAC replacement, surpassing the reduction in CO_2_ emissions (Fig. 4a). Also, RAC replacement contributes to significant stability improvement, especially when facing ECY conditions, and this contribution becomes increasingly pronounced in the 2WS case.

Similar to CO_2_ emissions, most provinces gain significant cost reduction among weather scenarios through RAC replacement, especially in EWYs. Under the 20C case, RAC replacement brings the highest total cost reduction during EWYs, which achieves 4.2 and 7.1% in the LE and AE scenario, respectively, which is higher than the reduction in CO_2_ emissions. In TWYs and ECYs, the total cost reduction can arrive at least 2.1% in the AE scenario, and both weather conditions show a higher reduction level for that on CO_2_ emissions. However, most provinces experience significant CO_2_ emission reduction due to the low-carbon transition, and the costs associated with frequent startups and shutdowns of coal-fired units undermine the magnitude of cost reductions. In the 2WS case, RAC replacement in the LE and AE scenarios achieves 1.2 and 2.6% cost reductions, respectively, in TWYs. A relatively great cost-saving effect is observed in extreme warm weather. Overall, RAC replacement in the LE and AE scenarios yields 3.7 and 6.4% cost savings in EWYs and 0.6 and 2.2% cost savings in ECYs, respectively. Chongqing, Hubei, Hunan, which suffer from heavy peak load pressure, get great cost reduction effects by the RAC replacement. In EWYs, Hunan achieves at most 17.8% cost reduction under 20C case, and arrive 21.0% under 2WS case, which effectively relief the power system. Moreover, under the 4WS case, RAC replacement achieves even more notable cost savings (Fig. S5b).

Loss of load probability (LOLP) is a main metric that evaluates the power system reliability; it happens when the electric loads exceed the existing generation capacities (45, 46). The replacement of RACs significantly reduces LOLP, particularly under ECY conditions, and this effect becomes more pronounced as the power system transitions toward low-carbon energy sources (Fig. 4b–f). Under the 20C case, no provinces experience load loss during TWYs or EWYs. In ECY conditions, however, a 1.0% LOLP is observed at the aggregate level under the NR replacement scenario. The LOLP levels are notably higher when accounting for the reduction of wind and solar generation (Fig. S6). RAC replacement significantly reduces the LOLP in both the LE and the AE scenarios, with the AE scenario successfully eliminating the loss of power supply in Chongqing. In Sichuan, the implementation of the AE scenario for RAC replacement substantially reliefs the threat of hydropower generation shortage, which decreases the LOLP from 8.5 to 6.5% (Fig. 4f). In the 2WS case, certain provinces experience load losses in the TWY and EWY conditions, exacerbating the ECY conditions. Chongqing and Hunan could achieve no LOLP by RAC replacement in TWYs and EWYs, respectively. In ECYs, Hubei and Guangxi successfully reduce the LOLP by 0.1% in the AE scenario, while Sichuan shows higher LOLP reduction by 2.1%. Compared with the 20C case, these provinces face more pronounced load losses while achieving better LOLP reductions through RAC replacement. With the further expansion of unstable renewable energy capacity in the 4WS case, provinces exhibit higher LOLP levels than in the 2WS case. However, RAC replacement continues to demonstrate greater LOLP reductions, particularly under ECY conditions (Fig. S5c–e).

### Comparative efficacy of RAC replacement versus battery storage

We incorporate battery storage capacities into the power system (AE–S scenarios) to evaluate the potential impact of RAC replacement. Storage technologies are essential for ensuring a stable power supply in systems with high renewable energy integration, enabling power shifting from valley load periods to peak hours. Unlike power supply shifting, RAC replacement directly reduces load. AE–S scenarios, which include supplementary storage capacities, are assessed alongside AE scenarios. Battery storage capacities are estimated based on the selling price and quantity of new RAC models, designed to meet the loads in the NR scenario (parameters in Tables S5 and S6) (41).

**Fig. 5.**
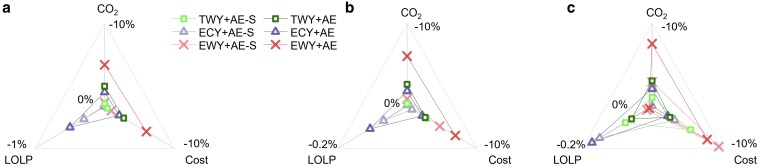
The difference between RAC replacement and adding battery storage. The figure shows annual CO_2_ emissions reduction, system cost saving, and LOLP reduction differences between RAC replacement and battery storage in all provinces of the southern China among weather scenarios and capacity cases. The coordinate axes use reverse-order scale values, leaning out to represent increasing emission reductions, cost savings, and reduced LOLP effect. The capacity cases for each subgraph are as follows: (a) 20C case; (b) 2WS case; (c) 4WS case.

Under the 20C case, RAC replacement demonstrates greater potential for addressing extreme weather events than battery storage, evidenced by larger reductions in CO_2_ emissions, costs, and LOLP. While battery storage achieves 0.3% emission reductions and 0.4% cost reductions in TWYs, the reductions are 2.4 and 2.7%, respectively for RAC replacement (Fig. 5a). This advantage widens under extreme weather conditions, with RAC replacement outperforming storage by 4.5 and 5.0% in EWYs and 1.6 and 1.8% in ECYs. Notably, in ECYs, RAC replacement reduces the LOLP from 0.99 to 0.84% in the AE scenario, versus only 0.90% for battery storage.

In the 2WS case, RAC replacement continues to outperform storage in reducing CO_2_ (43) emissions, costs, and LOLP under most weather conditions. However, in the 4WS case, with increased renewable electricity, battery storage performs much better in cost savings. In spite of this, RAC replacement consistently shows higher CO_2_ emission reductions (Fig. 5b). During peak loads, stored clean electricity is insufficient to meet demand, whereas load reduction through RAC replacement reduces reliance on thermal units.

For LOLP reduction, RAC replacement is more effective in ECYs, while battery storage shows slight advantages in TWYs and EWYs under the 4WS case. The limited renewable energy generation in ECYs makes load reduction more impactful. Under the 20C and 2WS cases, RAC replacement achieves 0.06% greater LOLP reductions than storage in ECYs (Fig. 5a and b). This advantage narrows to 0.03% in ECYs under the 4WS case. Conversely, in TWYs and EWYs under the 4WS case, storage achieves 0.02 and 0.01% greater reductions than RAC replacement, respectively, and the advance narrows to 0.03% in the ECY scenario under the 4WS case. However, with high renewable energy capacities under 4WS case, storage shows 0.02 and 0.01% greater reduction than RACs in TWYs and EWYs.

### Positive role of RAC replacement in tackling drought weather

**Fig. 6.**
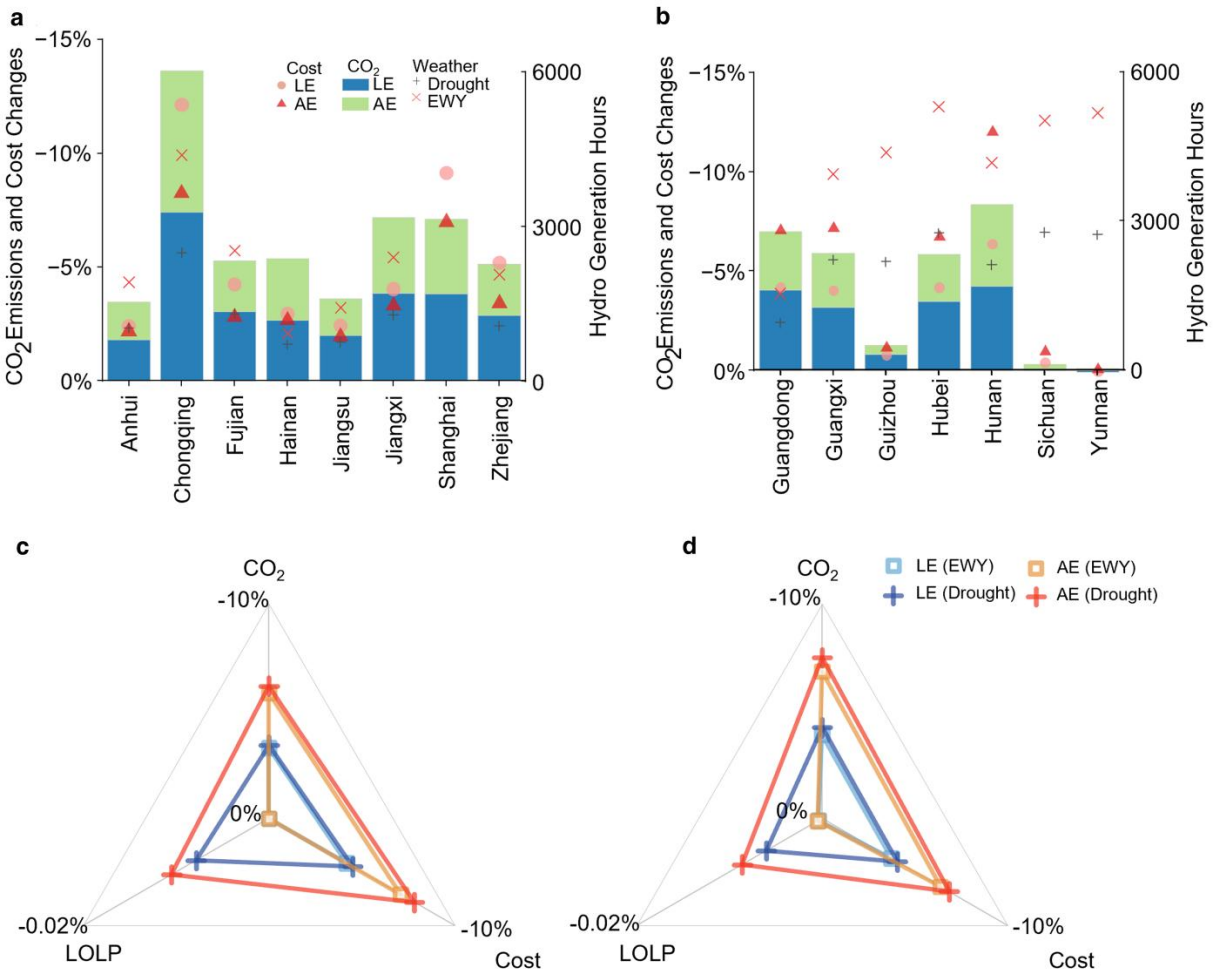
The role of RAC replacement in extreme warm and drought weather. a and b) The bar and scatter charts illustrate variations in annual CO_2_ emissions and cost implications during extreme warm and drought weather across provinces categorized by hydropower capacities—below 15,000MW (a) and above 15,000MW (b). The baseline values are derived from the EWY conditions under NR scenarios with the 20C case for each province. Cross points on the charts represent annual hydropower generation hours under different weather conditions. c and d) The figure illustrates the annual CO_2_ emissions, system costs, and LOLP during drought weather in the southern China within the 20C (c) and 2WS (d) capacity cases, relative to the values observed in EWY weather under NR scenarios within the same capacity case.

To estimate the RAC replacement effectiveness among various weather conditions, we construct extreme warm and drought weather conditions, as a supplementary scenario of EWYs. Compared with the EWY scenario, the RAC replacement scenario demonstrates greater CO_2_ emission reductions and cost savings during the drought weather scenario in the 20C case (Fig. 6). The ambient temperature has multiple effects on the hydropower output. With increasing global temperatures, precipitation levels across China tend to increase (43, 44, 47). In EWYs, precipitation significantly increases. However, high ambient temperatures may trigger extreme drought events, thereby reducing hydropower production (35, 48, 49). Provinces heavily reliant on hydropower experience staggering increases ranging from 41.1 to 133.9% in CO_2_ emissions and 34.2 to 130.9% in system costs compared with the EWY level under the 20C case (Fig. 6b). When facing drought, RAC replacement reduces CO_2_ emissions by a maximum of 13.7% and cost savings by a maximum of 18.4% among provinces; these values are 10.4 and 17.8%, respectively, in EWYs. Under the 20C scenario, at a total level, RAC replacement contributes at most 6.2% reduction in CO_2_ emissions and 7.8% reduction in total cost (Fig. 6c). However, with the retirement of stable coal-fired units in the 2WS case, the power system grapples with increased costs to meet rising demands during heat waves (Fig. 6d). Compared with that in the 20C case, in the 2WS case, the impact of CO_2_ emission reduction increases to 7.5%, while the cost-saving effect slightly decreases to 6.8% in the AE scenario. Notably, RAC replacement also strengthens power system stability under drought weather conditions. Moreover, RAC replacement reduces the LOLP from 0.012 to 0.002% in the 20C case and decreases it from 0.015 to 0.006% in the 2WS case.

## Discussion

The replacement of outdated RACs with more energy-efficient units significantly contributes to alleviating power loads, reducing CO_2_ emissions, lowering power system costs, and enhancing power system reliability. These benefits are evident not only in TWYs but also in extreme scenarios such as ECYs, EWYs, and droughts. The improvements are particularly noteworthy during ECYs, where they help offset the decline in renewable energy output that often accompanies these conditions. In EWYs, the increased use of RACs leads to higher peak loads, but the replacement of these appliances shows significant load reduction effects, resulting in substantial reductions in CO_2_ emissions and cost savings.

RAC replacement significantly reduces the peak power load by a maximum of 10.2% in TWYs. This effect becomes further pronounced during extreme weather events, with peak load ratio reductions reaching 13.5% during the ECY scenario. The load reduction effect is more pronounced for inland provinces than those in coastal regions. RAC replacement causes a varying decrease in CO_2_ emissions across extreme weather conditions. During the EWY, the decrease in CO_2_ emissions is more substantial than the decrease in load due to the increased integration of renewable energy generation. This phenomenon differs from the reduced reductions observed under ECY conditions, constraining renewable outputs. Furthermore, the reductions in costs resulting from RAC replacement surpass the reductions in CO_2_ emissions, particularly under extreme weather conditions. Under the dual challenges of extreme drought and warm weather, which adversely affect hydropower generation, RAC replacements offer a strong defense, resulting in greater emission and cost reductions during drought weather than in the EWY scenarios alone. These economic benefits are associated with enhanced power grid stability, as indicated by the reduced LOLP, particularly under ECY conditions. To achieve a relatively clean power system by the 2WS and 4WS cases, the advantages of RAC replacement become even more pronounced relative to the 2020 capacity baseline (the 20C case).

To assess the impact of RAC replacement, we compare its effectiveness with that of another option for enhancing system reliability, i.e. the battery storage system. Our analysis reveals that RAC replacement is more effective for power systems across various weather scenarios in the 20C case. Despite the potential for cost savings with battery storage in low-carbon power systems, RAC replacement consistently outperforms it in terms of emission reduction and power system stability.

It is argued that replacing outdated RACs can act as a key strategy for achieving carbon neutrality and enhancing power system reliability. The electricity demand for RACs is projected to increase significantly in the future due to rising ownership, as China remains the world's largest air-conditioner market, and higher ambient temperatures driven by climate change. Additionally, climate change impacts the cooling and heating demand of the tertiary sector, compounding the strain on the power system during peak load periods. Meeting these growing demands is becoming increasingly challenging as fluctuating renewable energy sources replace stable coal-fired power plants, while rising temperatures exacerbate the strain on thermal power units' generation and cooling systems, further limiting power supply. Moreover, improving energy efficiency in the tertiary sector is often more challenging and costly compared with residential sectors (50). Our research emphasizes the critical need for residents' adaptation of electricity consumption patterns to weather variations, particularly considering how temperature affects renewable energy generation and power transmission.

During RAC replacements, proper recycling of refrigerants from retired devices is essential to prevent their release into the atmosphere^a^ (51, 52). With the implementation of the national standard for refrigerant recycling in 2020, the target recycling rate is set to reach 90% (53). Trade-in policies are therefore an effective approach to subsidize households for RAC replacements while ensuring that retired devices are processed under standardized recycling protocols. Urban areas in southern China, where RACs were first widely adopted, have already demonstrated the benefits of this approach. Our findings offer a feasible path for other regions, including northern China, rural areas in southern China and other countries, where the growing use of RACs highlights the significant potential for further energy efficiency improvements through targeted replacement strategies.

Findings and insights in this work provide a forward-looking framework for trade-in policy in China (54), emphasizing their potential to mitigate the adverse impacts of extreme weather while serving as an effective demand-side strategy to enhance power system resilience. To further promote the adoption of high-efficiency models, the government could implement higher subsidy levels for high-efficiency equipment within the trade-in program. Meanwhile, optimizing the residential electricity tariff structure—such as through refined tiered tariffs or market-based pricing—could increase the operating costs for low-efficiency RACs, thereby encouraging their replacement, while having minimal impact on households already using high-efficiency models.

## Materials and methods

### Weather scenario construction

We employed hourly city-level weather data spanning three decades (1992–2022) to simulate domestic energy consumption and renewable energy generation in southern Chinese provinces (55, 56). Leap day data were removed to standardize each year to 8,760 h. The TWY scenario was constructed using the 30-year average values for hourly temperature, precipitation, wind speed, and solar radiation (Fig. S7). The ECY scenario was created with 8,760 h of weather data, selecting the lowest hourly temperature for each hour across 30 years (Fig. S8). Similarly, the EWY scenario was created using the warmest hourly temperatures over the same period (Fig. S9). For the ECY and EWY scenarios, the other weather variables (precipitation, wind speed, and solar radiation) were selected from the data corresponding to the same hours as the chosen temperature data to maintain climate consistency.

### Heating and cooling load simulation

A bottom-up approach was adopted to estimate the heating and cooling loads under various weather scenarios (Note S1), considering factors such as energy efficiency changes, RAC sales across different types, ambient temperature, residents' income, and room occupancy (Fig. S1). We analyzed the energy performance standards for two main types of RACs—fixed-speed, inverters (including cooling-only and heat pump models)—since 2000. Energy efficiency metrics were converted to APF for calculations. Using the RAC ownership data from the China Statistical Yearbook 2023, we treat the annual change in ownership as the sales for that year (11). Then, we introduced the typical APF (TAPF) in Eq. 1, which reflects consumer choices among AC types and energy efficiency grades, and the accumulated WAPF in Eq. 2, which represents the average efficiency of all existing RACs.


(1) $$\mathrm{TAP}F_{y}=\;\overset{5}{\underset{g=1}{\sum }}\;P_{y}^{f}\cdot \mathrm{grad}e_{g}\cdot \mathrm{AP}F_{\;f,y,g}$$

(2) $$\mathrm{WAP}F_{y,p,c}=\;\frac{\sum_{l=1}^{y}\;\mathrm{TAP}F_{l}\cdot AC_{l,p,c}}{\sum_{l=1}^{y}\;AC_{l,p,c}}$$

where $\mathrm{TAP}F_{y}$ is the TAPF in year *y*, *f* is the type of RAC, where 1 corresponds to fixed-speed type and 2 corresponds to inverter type, $P_{y}^{f}$ is the distribution of different types of RAC sales in year *y* (57), $\mathrm{grad}e_{g}$ is the distribution of RAC ownership across different grades, where the respective shares for grades 1 to 5 are 21, 18, 21, 20, and 21%, respectively (8). $\mathrm{AP}F_{\;f,y,g}$ is the energy efficiency levels of *f* type RACs of grade *g*, which comply with the latest published energy efficiency limit values in year *y*.

We estimated the electricity consumption of RAC systems by analyzing the relationship between ambient temperature and variations in urban household electricity use. The modeling tool for this analysis was a U-shaped piecewise function (Eq. 3), which synthesizes data from over 800,000 households in Shanghai collected between 2014 and 2016 (14). This function could provide a consistent pattern of household energy consumption while accounting for differences in the income and thermal efficiency of residences. A key assumption in our study was that no RAC systems were replaced prior to 2021, which could have led to an overestimation of the impact of RAC replacement. However, the function calculation period suggested that replacements occurring before 2016 would have minimal influence on our results. The function was segmented by temperature to reflect behavioral changes and RAC efficiency variations in response to temperature shifts.


(3) $${\mathrm{Eload}}_{y,t,p,c}^{\mathrm{SH}}=L^{\min}(k_{\mathrm{temp}}^{\mathrm{SH}}\cdot \mathrm{Tem}p_{y,t,p,c}+b_{\mathrm{temp}}^{\mathrm{SH}}-1)$$

where ${\mathrm{Eload}}_{y,t,p,c}^{\mathrm{SH}}$ is the electricity consumption attributed to household heating and cooling activities, considering the energy efficiency level and the prevalence of RAC ownership, within the context of Shanghai during the specified time frame, $\mathrm{Tem}p_{y,t,p,c}$ is the hourly ambient temperature under a weather scenario, $L^{\min}$ is the lowest electricity consumption level facing temperature variation, which we regard as the electricity consumption of neither heating nor cooling device operation, $k_{\mathrm{temp}}^{\mathrm{SH}}$ and $b_{\mathrm{temp}}^{\mathrm{SH}}$ are the slope and intercept of the temperature-response function, respectively, which is divided into six sections delineated by temperatures of 7, 13, 18, 20, and 25 °C (Fig. S2) (14).

Apart from RAC systems, distributed electric heaters (fan heaters, electric blankets, and infrared heaters) are widely used in southern China (8). To accurately estimate the heating electricity consumption of RAC systems, we excluded the energy consumption of distributed electric heaters accounted for in the piecewise function.


(4) $$P^{h}=\mathrm{htype}+(1-\mathrm{htype})\;\frac{\mathrm{WAP}F_{2015,1,1}}{\mathrm{EHefficiency}}$$

where $P^{h}$ is the proportion of households using ACs for room heating within the total urban household heating electricity consumption, htype is the proportion of households utilizing ACs for room heating, which is 63% (13), and EHefficiency is the energy efficiency of distributed electric heaters, which is 99% (58). In Eq. 5, we introduced the adjustment parameter $\alpha_{y,p,c}$ to address the WAPF gap between Shanghai and other cities.


(5) $$\alpha_{y,p,c}=\;\frac{\mathrm{WAP}F_{2015,1,1}}{O_{2015,1}\mathrm{WAP}F_{y,p,c}}$$

where $\alpha_{y,p,c}$ is the adjustment parameter of household RAC electricity consumption in year *y* within city *c* of province *p*, $\mathrm{WAP}F_{2015,1,1}$ is the WAPF in Shanghai in 2015, and $O_{2015,1}$ is the per household RAC ownership in year *y* in Shanghai. Correspondingly, we can obtain the heating and cooling load by RAC. $\mathrm{ACloa}d_{y,t,p,c}$ and the heating load using other electric heaters $\mathrm{EHloa}d_{y,t,p,c}$ via Eqs. 6 and 7, respectively. The determination of whether heating or cooling is necessary within households was guided by ambient temperatures. The cooling and heating degree day method typically uses a three-piece demand function, with distinct thresholds for heating and cooling and zero demand in between.

To better capture residents' energy consumption behavior, we applied a five-piece function. As shown in Fig. S2, this approach reflects minor demand changes in the 13 to 25 °C range, with gentler slopes for mild temperatures and steeper slopes for temperatures below 13 °C and above 25 °C. Twenty degree celsius is the turning point of the piece function, which is also consistent with the average value of “Indoor Air Quality Standard” recognized by the Chinese government. Nevertheless, this function could not capture all behavioral differences and immediate fluctuations in energy efficiency associated with these processes. Particularly during periods of extreme weather, both the actual energy efficiency of RAC systems and the resident usage patterns were likely to deviate from the norm. While the U-shaped piecewise load function indicated significantly increased electricity consumption under extreme temperature conditions, it did not completely represent the complex dynamics that occurred during these periods.


(6) $$\mathrm{ACloa}d_{y,t,p,c}=\{\begin{array}{cc} P^{h}\alpha_{y,p,c}\cdot AC_{y,p,c}\cdot {\mathrm{Eload}}_{y,t,p,c}^{SH}, & \mathrm{Temp}<20^{\circ }C\\ \alpha_{y,p,c}\cdot AC_{y,p,c}\cdot {\mathrm{Eload}}_{y,t,p,c}^{\mathrm{SH}},\; & \mathrm{else} \end{array}$$

(7) $$\mathrm{EHloa}d_{y,t,p,c}=(1-P^{h})AC_{y,p,c}\cdot {\mathrm{Eload}}_{y,t,p,c}^{\mathrm{SH}},\;\;\mathrm{Temp}<20^{\circ }C$$

To account for provincial differences in electricity consumption, we considered that local per capita GDP is a key driver of electricity usage. An adjustment factor, ϕ=0.477%, indicates that for every 1% increase in per capita GDP, residential electricity consumption rises by *ϕ* percentage points (59). This relationship allows us to adjust the electricity consumption of different provinces relative to that of Shanghai.


(8) $$\mathrm{HCloa}{d^{\prime }}_{y,t,p,c}=\{\begin{array}{c} \left(\phi \cdot \frac{\mathrm{PGD}P_{p}}{\mathrm{PGD}P_{\mathrm{SH}}}-\phi +1\right)\cdot \mathrm{HCloa}d_{y,t,p,c},\mathrm{PGD}P_{p}>\mathrm{PGD}P_{\mathrm{SH}}\\ \left(\frac{\mathrm{PGD}P_{p}/\mathrm{PGD}P_{\mathrm{SH}}}{\phi -(\phi -1)\cdot \mathrm{PGD}P_{p}/\mathrm{PGD}P_{\mathrm{SH}}}\right)\cdot \mathrm{HCloa}d_{y,t,p,c},\mathrm{PGD}P_{p}\leq \mathrm{PGD}P_{\mathrm{SH}} \end{array}$$

where $\mathrm{HCloa}{d^{\prime }}_{y,t,p,c}$ is the provincial heating and cooling load adjusted by income level, $\mathrm{PGD}P_{p}$ is the GDP per AC of *p* province in 2015, and $\mathrm{PGD}P_{\mathrm{SH}}$ is the GDP per capita of Shanghai in 2015.

We also consider the issue of residential occupancy rates. We selected the hourly occupancy rates in Chengdu as a reference for cities in southern China and made the following adjustments to the occupancy rates in our paper (60):


(9) $$\begin{array}{c} \mathrm{HCloa}{d^{\prime \prime }}_{y,t,p,c}=\;\frac{\mathrm{HCloa}{d^{\prime }}_{y,t,p,c}\cdot {\mathrm{Occupancy}}_{t}^{\mathrm{CD}}}{\mathrm{Occupanc}y^{\mathrm{avg}}}\;\;\; \end{array}$$

where $\mathrm{HCloa}{d^{\prime \prime }}_{y,t,p,c}$ represents the electricity consumption adjusted based on hourly occupancy rates and income level, $\mathrm{Occupanc}y^{\mathrm{avg}}$ refers to the average hourly occupancy rate across a 24-h period in Chengdu, and ${\mathrm{Occupancy}}_{t}^{\mathrm{CD}}$ represents the hourly occupancy rate in Chengdu per day (Fig. S3).

To estimate the contribution of RAC energy efficiency improvement, we formulated scenarios considering the absence of RAC replacements before 2021 and two replacement scenarios for RAC systems before 2009 (the year that at least one version of the energy efficiency label was applied to two types of RACs). The replacement scenarios involved upgrading these older RAC systems to the most recent energy efficiency level type (40). The LE scenario assumed that residents replaced their RAC with the LE in 2021. In the AE scenario, residents were assumed to have replaced their RAC with the AE in 2021, and residents used only this RAC system for room heating.

### Power economic dispatch under extreme weather conditions

A multiregional economic power dispatch model was developed to evaluate the effects of extreme weather on power systems. The model incorporated data on power generation capacity, fuel consumption rates, interregional transmission, and power loads (Note S2). We assumed constant power transmission with provinces outside southern China based on the annual cross-province electricity transmission data from 2020 (61). The model encompassed seven types of energy technologies: coal, natural gas, biomass, nuclear, hydro, wind, and solar. In addition, the model included pumped storage.

Nuclear power generation was considered constant (nondispatchable), following the full-load hours recorded in 2020 (61). Weather-dependent capacity factors for renewable technologies, including wind, solar, and hydropower generation, were calculated based on the specific weather conditions they require. Wind power was determined using wind speed and ambient temperature. For solar energy, capacity factors were calculated using solar radiation and temperature. Regional weather patterns under different conditions are presented in Figs. S7–S9, and the formulas for capacity factors are provided in Note S2. Hydropower generation was calculated on a monthly basis, considering ambient temperature, precipitation, and hydropower feed-in tariffs, as described in Note S3. Additionally, we incorporated a 4-h lithium-ion battery storage system to evaluate the effectiveness of RAC replacement under the AE scenario, noting that this storage technology required ∼5 h for a full charge and could discharge a maximum of 25% of its 4-h capacity per hour (the calculation of battery storage capacities is detailed in Note S2) (41).

Hourly electric loads for each province in 2018 were analyzed, factoring in urban household heating and cooling loads under that year's weather conditions. We derived the base power loads by calculating heating and cooling demands using Eqs. 1–9 and subtracting them from the provincial loads. These base loads were then modified by adding residential heating and cooling demands to simulate total loads under various RAC replacement and weather conditions, which were subsequently used to project renewable energy generation.

Three scenarios were established to simulate the transition toward an increasingly sustainable power system: the 20C case, 2WS case, and 4WS case. In the 2WS and 4WS case, the wind and solar generation capacities were projected to double and quadruple wind and solar capacities doubled relative to 2020 levels, coinciding with the phasing out of coal-fired power plants (Note S4). To compensate for the variability in renewable energy generation, we calculated the equivalent renewable energy capacity by multiplying the additional installed capacity by the annual utilization hours of wind and solar power for each province in 2020. This equivalent capacity was designed to replace a corresponding quantity of the maximum coal-fired generation capacity. The optimization model was solved using the CPLEX solver within the general algebraic modeling system (GAMS) platform.

The impacts of RAC systems were measured in terms of their contributions to CO_2_ emissions, system cost reductions, and system stability changes. CO_2_ emissions and power system costs were computed from the perspective of electricity consumption (Note S5). Transmission losses were integrated into the emissions and cost calculations. System reliability was assessed using the LOLP metric, as outlined in Eq. 10.


(10) $$\begin{array}{c} \mathrm{LOL}P_{p}=\;\mathrm{Max}\left(\frac{\sum_{t\in \Phi^{T}}\;\mathrm{LP}S_{t,p}}{\sum_{t\in \Phi^{T}}\;L_{t,p}^{\mathrm{Total}}},0\right)\; \end{array}$$

where $\mathrm{LP}S_{t,p}$ is the loss of power supply for province *p*, which is equal to the difference between the available generation capacity and loads, and $L_{t,p}^{\mathrm{Total}}$ is the total power load containing the residential heating and cooling demand.

## Supplementary Material

pgaf230_Supplementary_Data

## Data Availability

Weather data are available in the ERA5 dataset from the European Centre for Medium-Range Weather Forecasts (https://www.ecmwf.int/en/forecasts/dataset/ecmwf-reanalysis-v5). Urban RAC ownership data are calculated based on the main durable goods owned per 100 urban households and household data, both sourced from the National Statistical Yearbook published by the National Bureau of Statistics of China (https://www.stats.gov.cn/sj/ndsj/2023/indexeh.htm). Changes in China's energy-efficiency label grade thresholds can be found by their file numbers listed in Supplementary material (https://www.chinesestandard.net/). Population data are accessible through LandScan (https://landscan.ornl.gov/). The remaining data required to evaluate the conclusions of the paper are included in the paper and its Supplementary material. Parameter settings of different technologies are shown in Table S3 and emission factors, fuel cost, and installed capacities in different capacity cases are presented in Tables S7–S9. The codes for data processing are conducted in Matlab (R2023b) and GAMS 25.1.1, and data illustration is generated in Origin 2024 and ArcGIS 10.8.

## Note

a Ozone-depleting substances, such as Freon 22 (R22) and hydro-chlorofluorocarbons (HCFCs), were widely used as refrigerants in outdated RACs. Under the Montreal Protocol, China declears to phase out ozone-depleting substances by 2030, and newer RAC models will use environmentally friendly refrigerants.
