# Supplementary material for: Air-conditioning replacement to enhance the reliability of renewable power systems under extreme weather risks
Source: PNAS Nexus. 2025 Jul 23;4(8):pgaf230. doi: 10.1093/pnasnexus/pgaf230 (PMC12342146; doi:10.1093/pnasnexus/pgaf230)
Supplement: pgaf230_Supplementary_Data [file pgaf230_supplementary_data.pdf]

Supplementary Information *for*

Air-Conditioning Replacement to Enhance the Reliability of Renewable Power Systems Under Extreme Weather Risks

The supplementary file includes:

Supplementary Note 1-5

Supplementary Table 1-9

Supplementary Figure 1-9

Supplementary References

Supplementary Note 1. Residents Load Simulation

1.1 China's Room Air Conditioning Energy Efficiency Standards

Room air conditioning energy efficiency measures the energy used to provide a specific cooling or heating capacity. To encourage energy efficiency and guide consumers toward energy-saving products, China issued its first energy performance standards for RACs in 2000. Between 2000 and 2021, China introduced six versions of these standards, as detailed in Supplementary Table 1. These standards classify RACs based on their energy efficiency labels, typically ranging across five distinct grades. Grade 5 represents the minimum energy performance level, while Grade 1 signifies the most efficient products.

Split-type air conditioning, specifically fixed-speed air conditioning (FAC) and inverter air conditioning (IAC) are the prevailing types of RACs. GB12021.3 restricts the energy efficiency of FACs, whereas GB21455 regulates IACs efficiency. Notably, GB21455-2019 addresses both FACs and IACs. Energy efficiency metrics vary across RAC types. For FAC, the Energy Efficiency Ratio (EER) determines efficiency by comparing rated cooling capacity (CC) to rated cooling power consumption. Over the years, four distinct FAC standards emerged, progressively increasing the minimum EER from 2.5 to 3.7 by 2019.

In contrast to FACs, IACs maintain room temperature stability by adjusting compressor speeds, offering energy conservation benefits. IAC adoption in China is relatively recent, with the first energy efficiency standard introduced in 2008. From 2013, IACs were divided into cooling-only products and heat pumps. Cooling-only units are rated using seasonal energy efficiency ratio (SEER), while heat pumps utilize an annual performance factor (APF). By 2019, the minimum SEER had risen from 3 to 3.7, and the APF had reached 3.3. In 2022, China introduced the advanced energy efficiency level of RACs with an APF of 6 to promote the use of energy-saving products(1).

1.2 Residents Heating and Cooling Model

We constructed a city residents' heating and cooling load simulation model using a bottom-up approach, considering factors such as energy efficiency changes, RAC sales across different types, ambient temperature, residents' income, and house occupancy (Supplementary Fig. 1). Nonetheless, weather conditions can escalate electricity demand in industrial sectors, public buildings, and rural

homes, potentially leading to increased peak loads. In this study, we primarily focus on the impact of urban residential consumption on electricity load.

Based on the energy efficiency standard update, we get the energy efficiency of RACs purchased in different years. Unlike seasonal metrics like SEER and APF, EER does not account for ambient temperature or part-load operation effects on energy efficiency. Moreover, while EER and SEER consider cooling efficiency, they lack consideration for heating efficiency. To address this, SEER and EER are converted into APF to gauge changes in energy performance standards. The relationship between EER and APF is shown in Eq. (1)(2, 3).

$$APF_{f,y,g} = 0.707 \times EER_{f,y,g} + 0.43 \tag{1}$$

f denotes the type of RACs, where 1 corresponds to FAC and 2 corresponds to IAC. The variable y represents the year, while g denotes the energy label of RACs, with $g \epsilon \{1,2,3,4,5\}$. $APF_{f,y,g}$ and $EER_{f,y,g}$ show the energy efficiency level of f type RACs with grade g, which comply the latest published energy efficiency limited values in year y.

For the cooling-only type, SEER was converted into EER and then into APF using Eq. (2).

$$EER_{2,y,g} = 0.988 \times SEER_{2008,g} \tag{2}$$

$$AC_{y,p,c} = UH_{y,p,c} \cdot O_{y,p} - UH_{y-1,p,c} \cdot O_{y-1,p} \tag{3}$$

Eq. (3) represents RAC sales volume, denoted as $AC_{y,p,c}$, in year y within city c of province p. Urban household $UH_{y,p,c}$ was calculated as the quotient of urban population and household size, using population data from LandScan for city population and China's national population census for household size(4, 5). $O_{y,p}$ signifies the per household ownership of RACs in year y of province p.

To capture detailed consumer preferences in purchasing, we introduce the parameter typical annual performance factor (TAPF) in Eq. (4), reflecting the APF value that encompasses consumer choices among AC types and grades.

$$TAPF_y = \sum_{g=1}^{5} P_y^f \cdot grade_g \cdot APF_{f,y,g} \tag{4}$$

$TAPF_y$ show the TAPF in year y, P_y^f signifies the distribution of FAC and IAC sales(3) in year y. $grade_g$ represents the distribution of RAC ownership across different grades, where the respective shares for grades 1 to 5 are 21%, 18%, 21%, 20%, and 21%(6).

The evolution of energy performance standards has resulted in significant disparities in the energy efficiency of RACs purchased in different years. To account for the influence of these standard updates, we introduce the concept of weighted annual performance factor (WAPF), representing the average efficiency level of all existing RACs in year y, as depicted in Eq. (5). Notably, in 2013, the National Bureau of Statistics of China revised its urban and rural survey methodology. As a result, it wasn't until 2016 that per household ownership of RACs surpassed 2012 levels. To avoid bias from these methodological changes, we kept the WAPF values for 2013 to 2015 consistent with the 2012 value. This adjustment does not impact the effectiveness of the RAC replacement analysis, as the focus is on replacing outdated RACs manufactured before 2009, which the changes after 2012 do not influence.

$$WAPF_{y,p,c} = \frac{\sum_{l=1}^{y} TAPF_l \cdot AC_{l,p,c}}{\sum_{l=1}^{y} AC_{l,p,c}} \tag{5}$$

Estimating hourly electricity consumption of RACs involves complexity due to multifaceted dependencies, including ambient temperature, household occupancy, thermal insulation, personal income and educational levels(6). To estimate RAC electricity consumption, we leverage the correlation between ambient temperature and urban household electricity usage variation. A U-shaped piecewise function, based on data from over 800,000 residences in Shanghai between 2014 and 2016(7), was established and is depicted in Supplementary Fig. 2.

And we have:

$$Eload_{y,t,p,c}^{SH} = L^{min}\left(k_{temp}^{SH} \cdot Temp_{y,t,p,c} + b_{temp}^{SH} - 1\right) \tag{6}$$

$Eload_{y,t,p,c}^{SH}$ denotes the mean electricity consumption attributed to household heating and cooling activities, accounting for both the energy efficiency level and the ownership of RACs, within the context of Shanghai during the specified time frame. $Temp_{y,t,p,c}$ is hourly ambient temperature under a weather condition. At 20 °C, denoted as L^{min}, neither heating nor cooling devices operate, and hence this point is excluded from calculations. Parameters k_{temp}^{SH} and b_{temp}^{SH} represent the slope and intercept of temperature-respond function, dividing it into six sections delineated by temperatures 7 °C, 13 °C, 18 °C, 20 °C, and 25 °C, respectively.

In Eq. (7), we introduce the adjustment parameter $\alpha_{y,p,c}$ to address the WAPF gap between Shanghai and other cities.

$$\alpha_{y,p,c} = \frac{WAPF_{2015,1,1}}{O_{2015,1} WAPF_{y,p,c}} \tag{7}$$

$\alpha_{y,p,c}$ is the adjustment parameter of household RACs electricity consumption in year y within city c of province p. $WAPF_{2015,1,1}$ shows the WAPF level in Shanghai in 2015, reflecting the average APF level in Eq. (6). $O_{2015,1}$ represent the per household RACs ownership in year y in Shanghai.

The division of $WAPF_{2015,1,1}$ and $O_{2015,1}$ converts the electricity consumption into the corresponding capacity measurement for individual RAC units. Then, it converts to the electricity consumption by dividing it with regional heterogeneity WAPF level $WAPF_{y,p,c}$.

Because of the leak of district heating, electric heating has become primary for winter heating in southern China(8). Apart from RACs, distributed electric heaters (fan heaters, electric blankets, and infrared heaters) are also widely used in southern China. To estimate RACs' heating electricity consumption accurately, we exclude distributed electric heaters' energy consumption accounted for in the piecewise function.

$$P^h = htype + (1 - htype)\frac{WAPF_{2015,1,1}}{EHefficiency} \tag{8}$$

Eq. (8) introduces the proportion P^h of households using RACs for room heating within the total urban household heating electricity consumption. $htype$ denotes the proportion of households utilizing RACs for room heating, which is measured at 63%(8). $EHefficiency$ represents the energy efficiency of distributed electric heaters, established at 99%(9). Therefore, the piecewise RAC load function is shown in Eq. (9).

$$ACload_{y,t,p,c} = \begin{cases} P^h \alpha_{y,p,c} \cdot AC_{y,p,c} \cdot Eload^{SH}_{y,t,p,c}, Temp < 20℃ \\ \alpha_{y,p,c} \cdot AC_{y,p,c} \cdot Eload^{SH}_{y,t,p,c}, else \end{cases} \tag{9}$$

$ACload_{y,t,p,c}$ is the total RAC load in hour t with WAPF in year y within city c of province p. When the ambient temperature falls below 20°C, households opt for heating, while temperatures exceeding 20°C residents choose to cooling. Correspondingly we can also get the heating load using other electric heaters $EHload_{y,t,p,c}$ in Eq. (10).

$$EHload_{y,t,p,c} = (1 - P^h)AC_{y,p,c} \cdot Eload^{SH}_{y,t,p,c} \tag{10}$$

The widespread use of low efficiency distributed electric heaters can be attributed to various factors. Some individuals perceive these heaters to offer superior thermal comfort compared to RACs(10). Others resort to distributed electric heaters due to older RACs functioning solely for cooling purposes, making the use of these less efficient RACs for room heating considerably more expensive than employing distributed electric heaters. Upgrading these older RACs to heat pump

types presents an opportunity for residents to switch to RACs for heating, potentially resulting in reduced electricity bills(11). Under the AE scenario assumption, all residents exclusively use RACs for room heating. Eq. (11) illustrates the residential heating and cooling power load function.

$$HCload_{y,t,p,c} = \begin{cases} P^h \alpha_{y,p,c} + (1 - P^h)(\frac{EHefficiency}{WAPF_{y,p,c}}) \cdot AC_{y,p,c} \cdot Eload^{SH}_{y,t,p,c}, Temp < 20℃ \\ \alpha_{y,p,c} \cdot AC_{y,p,c} \cdot Eload^{SH}_{y,t,p,c}, else \end{cases} \tag{11}$$

To account for provincial differences in electricity consumption, we considered that local per capita GDP is a key driver of electricity usage. An adjustment factor, $\phi = 0.477\%$, indicates that for every 1% increase in per capita GDP, residential electricity consumption rises by ϕ percentage points(59). This relationship allows us to adjust the electricity consumption of different provinces relative to that of Shanghai.

$$HCload^{'}_{y,t,p,c} = \begin{cases} (\phi \cdot \frac{PGDP_p}{PGDP_{SH}} - \phi + 1) \cdot HCload_{y,t,p,c}, PGDP_p > PGDP_{SH} \\ (\frac{PGDP_p/PGDP_{SH}}{\phi - (\phi - 1) \cdot PGDP_p/PGDP_{SH}}) \cdot HCload_{y,t,p,c}, PGDP_p \le PGDP_{SH} \end{cases} \tag{12}$$

$HCload^{'}_{y,t,p,c}$ is the provincial heating and cooling load adjusted by income level, $PGDP_p$ is the GDP per AC of p province in 2015, and $PGDP_{SH}$ is the GDP per capita of Shanghai in 2015.

In order to consider the issue of residential occupancy rates, we selected the hourly occupancy rates in Chengdu as a reference for cities in Southern China, and made the following adjustments to the occupancy rates in our paper(13):

$$HCload^{''}_{y,t,p,c} = \frac{HCload'_{y,t,p,c} \cdot Occupancy^{CD}_t}{Occupancy^{avg}} \tag{13}$$

$HCload^{''}_{y,t,p,c}$ represents the electricity consumption adjusted based on hourly occupancy rates and income level, $Occupancy^{avg}$ refers to the average hourly occupancy rate across a 24-hour period in Chengdu, and $Occupancy^{CD}_t$ represents the hourly occupancy rate in Chengdu per day.

Supplementary Note 2. Inter-regional Power Dispatching Model

To assess the impact of RAC replacement on the power system, we developed a multiregional economic power dispatch model for southern China. RAC replacement reduces system load, leading to changes in power dispatch decisions that focus on cost savings. Consequently, CO_2 emissions also shift as thermal power generation is adjusted. By reducing cooling and heating loads, the power system gains more flexibility to handle potential peak load challenges, enhancing overall system resilience.

Given the scope of our research, all provinces in mainland China, except for the ones under study, were treated as external provinces. The research provinces and their regional affiliations are listed in Supplementary Table 2. The inter-regional power transmission was divided into cross-regional transmission between the research and external provinces and the transmission of the research provinces. The transmission between the research and external provinces was calculated by using the annual cross-province electricity transmission data in 2020(14). We averaged the power transmission between the external and research provinces to the hourly level and deemed it as the transmission capacity of these lines, making it a priority to meet the power demand. For research provinces, supercritical and ultra-supercritical power transmission are considered(15). To close the model, we set the loss of power supply (LPS), which is equal to the difference between the available generation capacity and loads, to estimate the amount of load losses.

As shown in Supplementary Table 2, the provinces in southern China belong to different regional power grids. Specifically, the East China Power Grid and Central China Power Grid are part of the State Grid Corporation of China, while the Southern China Power Grid operates independently. In our research, we assume that power transmission between these grids is not constrained by the market power or company boundaries. Instead, the power transmission decisions are based solely on minimizing the total system cost. This assumption allows us to model the power flow between regions as a function of economic efficiency rather than being limited by the administrative or corporate boundaries of the grid operators.

We construct the model to minimize the total cost of the power system under the constraint of meeting the hourly power loads. The model included seven types of energy technologies: coal, natural gas, biomass, nuclear, wind, solar, and hydro, along with pumped storage. Nuclear power

generation was assumed to remain constant (nondispatchable). Cost parameters for the other technologies are provided in Supplementary Table 3. The mathematical formulation for the objective function is as follows:

$$min\ F = \sum_{t\in\Phi^T}\sum_{o\in\Phi^O}\sum_{p\in\Phi^P} ge_{t,o,p} \cdot \left(fuelcost_{o,p} + OMcost_o\right) + st_{t,o,p} \cdot stcost_o \tag{14}$$

We considered the following power system costs: $fuelcost_o$, $OMcost_o$, and $stcost_o$ that denote the unit cost (Yuan/MWh) of technology o in the generator fuel, operation and maintenance, and generator start-up, respectively[10,14]. Φ^O is the set of generation technologies. The power generation (MWh) $ge_{t,o,p}$ and the start-up capacity (MW) $st_{t,o,p}$ are the decision variables of this model.

We collected the hourly provincial electric loads for the year 2018, which also included the urban household heating and cooling loads under the weather conditions of that year. Accordingly, we compute the heating and cooling loads using Eq. (13) and subtract it from the provincial loads to derive the base power loads $L_{t,p}^{b}$. For a certain year y, we can get the total power load containing residential heating and cooling demand $L_{t,p}^{Total}$ in Eq. (15).

$$L_{t,p}^{Total} = L_{t,p}^{b} + \sum\nolimits_{c\in C^P} HCload''_{y,t,p,c}, \forall t \in \Phi^T, \forall p \in \Phi^P \tag{15}$$

$$\sum_{o\in\Phi^O, p\in\Phi^P} ge_{t,o,p} - \sum_{p\in\Phi^P} ch_{t,sto,p} \geq \sum_{p\in\Phi^P} L_{t,p}^{Total}, \forall t \in \Phi^T \tag{16}$$

$$\sum_{o\in\Phi^O} ge_{t,o,p} + \sum_{q\in\Phi^P} \left(1 - loss_{q,p}\right) \cdot \left(1 - temploss_{t,p}\right) \cdot tr_{t,q,p} - tr_{t,p,q} - ch_{t,sto,p} - LPS_{t,p}$$
$$= L_{t,p}^{Total}, \forall t \in \Phi^T, \forall p \in \Phi^P \tag{17}$$

Eqs. (16) and (17) show the hourly balance that the power supply meets the load demand for southern China and p province, respectively. q also represents the province and is employed in calculating interregional power transmission. $loss_{p,q}$ is the transmission loss (%) between the p and q provinces. $temploss_{t,p}$ is the temperature effect on transmission loss (%). $tr_{t,p,q}$ is the inter-regional power transmission (MWh) from p to q province. $LPS_{t,p}$ is the LPS (MWh) for the p province. This variable aims to close the model, which has an extremely high cost that ensures it is called last in power dispatching. $ch_{t,sto,p}$ is the power charge (MWh) in the pumped storage.

To account for transmission system losses dependent on temperature, we calculated line losses associated with power across different temperature ranges. The method involves determining power losses at various capacities within each temperature range. A capacity-weighted average of these power losses is then computed to derive the average loss rate for each temperature range(16).

$$temploss_{t,p} = \begin{cases} 0.0146, Temp_{t,p} < 0℃ \\ 0, 0℃ \le Temp_{t,p} \le 10℃ \\ 0.0145, Temp_{t,p} > 10℃ \end{cases}, \forall t \in \Phi^T, \forall p \in \Phi^P \tag{18}$$

$temploss_{t,p}$ represents the power loss rate at the corresponding temperature.

$$tr_{t,p,q} \le tr_{p,q}^{line}, \forall t \in \Phi^T, \forall p, q \in \Phi^P \tag{19}$$

$$ge_{t-1,o,p} + st_{t,o,p} - sd_{t,o,p} = ge_{t,o,p}, \forall t \in \Phi^T, \forall o \in \Phi^O, \forall p \in \Phi^P \tag{20}$$

$$st_{t,o,p} \le capacity_{o,p} \cdot ramp_o, \forall t \in \Phi^T, \forall o \in \Phi^O, \forall p \in \Phi^P \tag{21}$$

$$sd_{t,o,p} \le capacity_{o,p} \cdot ramp_o, \forall t \in \Phi^T, \forall o \in \Phi^O, \forall p \in \Phi^P \tag{22}$$

$$ge_{t,o,p} \le capacity_{o,p} \cdot cf_o, \forall t \in \Phi^T, \forall o \in \Phi^O, \forall p \in \Phi^P \tag{23}$$

$$cf_{t,wind,p} = \begin{cases} 0 & v_{t,c} \le v_{in} \ or \ v_{t,c} \ge v_{out} \\ \frac{(k^{wind} Temp_{t,p} + b^{wind})(v_{t,p} - v_{in})}{v_{rated} - v_{in}} & v_{in} \le v_{t,c} \le v_{rated} \\ k^{wind} Temp_{t,p} + b^{wind} & else \end{cases}, \forall t \in \Phi^T, \forall p \in \Phi^P \tag{24}$$

$$cf_{t,solar,p} = \frac{G_{t,p}}{G_{STC}} \cdot \left[1 + \alpha\left(\left(Temp_{t,p} + (u \cdot G_{t,p})\right) - Temp^{STC}\right)\right], \forall t \in \Phi^T, \forall p \in \Phi^P \tag{25}$$

$$ch_{t,sto,p} \cdot ef_{sto} - ge_{t,sto,p} + stock_{t-1,sto,p} = stock_{t,sto,p}, \forall t \in \Phi^T, \forall sto \in \Phi^{STO}, \forall p \in \Phi^P \tag{26}$$

$$ch_{t,sto,p} \le capacity_{sto,p} \cdot SC_{sto}, \forall t \in \Phi^T, \forall sto \in \Phi^{STO}, \forall p \in \Phi^P \tag{27}$$

$$stock_{t,sto,p} \le capacity_{sto,p} \cdot SF_{sto}, \forall t \in \Phi^T, \forall sto \in \Phi^{STO}, \forall p \in \Phi^P \tag{28}$$

Eq. (19) is the transmission line capacity limit, in which $tr_{p,q}^{line}$ is the maximum transmission capacity (MW) between province p and q. Eqs. (19)-(22) shows the ramp constraints of the generation capacity, as shown in Supplementary Table 3 and 4. $st_{t,o,p}$ and $sd_{t,o,p}$ are the ramp-up and ramp-down capacity (MWh). $ramp_o$ is the ramp constraints of technology o.

Eq. (23) provide the maximum generation levels of the technologies. $capacity_{o,p}$ is the installed power capacity of the o technology in the p province. cf_o is the capacity factor of the o technology (Supplementary Table 3). We assume that nuclear power is non-dispatchable, with its generation determined by the full-load hours in 2020(14). Capacity factors are calculated individually for technologies influenced by weather conditions, including wind, solar, and hydro.

The capacity factor of the wind and solar is calculated using Eq. (24) and (25), with the resulting capacity factors for typical weather years presented in Supplementary Table 4. These weather data

are identical to the weather data in the heating and cooling load simulation. The generated power of the wind turbine is affected by the wind speed (m/s) $v_{n,c}$ and temperature (°C) $Temp_{t,p}$. v_{in}, v_{out}, and v_{rated} are the cut-in, cut-out, and rated speeds of the wind turbine, respectively. k^{wind} and b^{wind} show the influence of temperature on the wind turbine performance. Solar radiation $G_{t,p}$ and temperature $Temp_{t,p}$ determine the capacity factor of the solar power, G_{STC} and $Temp^{STC}$ denote the solar radiation and the temperature, respectively, under the standard condition. α denotes the ambient temperature coefficient. u is the temperature coefficient. The following parameters were used in the calculation: $v_{in} = 3\frac{m}{s}$; $v_{out} = 25\frac{m}{s}$; $v_{rated} = 13\frac{m}{s}$(17); $k^{wind} = 3.68 \times 10^{-3}$; $b^{wind} = 0.908$ (18); $G_{STC} = 1000\frac{W}{m^2}$; $Temp^{STC}$=25 °C; $\alpha = -3.7 \times 10^{-3}\left(\frac{1}{°C}\right); and\ u = 0.0256$(19).

Eq. (26) and (27) show the charge/discharge constraints for the storage technology sto, encompassing pumped storage across all scenarios and incorporating battery storage specifically for the AE–S scenarios. Within these equations, SF_{sto} is the maximum storage capacity of sto technology(20, 21). SC_{sto} is the maximum charge capacity of sto technology. ef_{sto} shows the round-trip efficiency of sto technology(20, 21). Equations (28) show the storage capacity constraint.

The installed capacity of battery storage is detailed in Supplementary Table 5, while Supplementary Table 6 provides the parameters of battery storage.The battery storage system modeled in this study has a 4-hour discharge and 5-hour charge duration. The reference configuration (0.25 MW, 1 MWh, 100% depth of discharge (DoD), 80% charging efficiency) was scaled to 1 MW with 25% DoD (1 MWh) and 20% charging efficiency to match the model's hourly resolution. These adjustments ensure functional and cost equivalence while enabling power regulation compatible with the hourly dispatching model.

Supplementary Note 3 Hydropower Generation Model

The capacity factor of the hydropower is calculated using Eq. (29)-(32). There are seasonal fluctuations in hydropower generation. In this paper, the logistic regression model is referenced to calculate the monthly hydropower capacity factor for the southern provinces. The paper uses cooling degree days (CDD) as a temperature-related variable.

$$CDD_{y,m,p} = \frac{1}{C_p}\sum_{c=1}^{C_p}\sum_{d=1}^{D_m}\overline{Temp}_{y,m,d,p,c} - T_R, \forall m \in [1,12], \forall p \in \Phi^P \quad (29)$$

As shown in Eq. (29), the CDD is the sum of the difference between the mean temperature and the reference temperature in a month. D_m is the number of days in month m, $\overline{Temp}_{y,m,d,p,c}$ is the mean temperature at day d in month m of year y within city c in province p, which calculated by the ambient temperature data. T_R is the reference temperature 17°C.

$$\ln{(HY)}_{y,m,p} = 0.128 lnCDD_{y,m,p} + 0.072 lnR_{y,m,p} + 0.205 lnCapacity_{y,hydro,p}$$
$$+0.272 lnPrice_{y,p} + 7.219 \quad (30)$$

Eq. (30) shows the logarithmic regression model for hydropower, encompassing the 95% confidence interval(22). The equation shows the monthly hydropower generation $HY_{y,m,p}$, which is influenced by several factors: temperature shown by $CDD_{y,m,p}$, precipitation $R_{y,m,p}$, installed hydropower capacity $Capacity_{y,hydro,p}$, and average hydropower feed-in tariff $Price_p$.

To calibrate the outcome of Eq. (30), we utilize the recorded hydropower generation data for each province in 2020. Equation (31) introduces the calibration parameter for hydropower ω_p derived as the ratio between the actual hydropower generation in 2020 and the computed value $HY_{2020,m,p}$. The actual hydropower generation is calculated using the capacity factor $CFhydro_p$ and capacity $Capacity_{hydro,p}$ of hydro power.

$$\omega_p = \frac{CFhydro_p \cdot Capacity_{hydro,p}}{\sum_{m=1}^{12} HY_{2020,m,p}}, \forall p \in \Phi^P \quad (31)$$

To specifically assess the impact of precipitation on generation, we will utilize precipitation data from the corresponding climate scenario and the temperature at the same hour in 2020. The hydropower capacity factors, as shown in Eq. (32), can be obtained based on the calibration parameter, with the maximum capacity factor limited to 0.8(23). $HYh_{t,p}$ is equal to $HY_{m,p}$ for the month in which it is situated.

$$cf_{t,hydro,p} = \min\left(\frac{\omega_p \cdot HYh_{t,p}}{Capacity_{hydro,p}}, 0.8\right), \forall t \in \Phi^T, \forall p \in \Phi^P \quad (32)$$

Supplementary Note 4. Capacity Case Setting

To simulate the development pathways of a clean power system, three scenarios were established: the 2020 capacity (20C) case, double (2WS) and quadruple (4WS) wind and solar capacities doubled relative to 2020 levels. Supplementary Table 7 presents the installed capacity of different technologies in the 20C case. Supplementary Table 8 illustrates the changes in installed capacity under the 2WS and 4WS case.

In the 2WS and 4WS cases, the installed capacities of wind and solar power are projected to double and quadruple, respectively, compared to the 20C scenario (11). Given that renewable energy generation is directly influenced by weather conditions, the actual power output from coal and renewable sources will differ even with the same installed capacity. We calculated the equivalent renewable energy generation capacity by multiplying the additional renewable capacity by the annual utilization hours of wind and solar power for each province in 2020. This equivalent capacity will replace the highest power generation capacity (95% of total capacity) in the scenarios. Consequently, the retirement of some coal-fired power units is proposed to meet the strategic goal of peak carbon emissions before 2030. Notably, the capacity targets were established in 2022, and with the rapid expansion of renewable energy, some provinces had already met the renewable energy capacities in 2WS and 4WS scenarios by the end of 2024.

Supplementary Note 5. CO_2 Emissions and Cost by the Consumer Side

CO_2 emissions by the power system are summed by the emissions from coal-fired and gas-fired power plants. The hourly CO_2 emissions produced by the province p is calculated as Eq. (33):

$$EP_{t,p} = o^C \, \lambda^C ge_{t,coal,p} \cdot a_p^C + e^G ge_{t,gas,p} \tag{33}$$

$$CP_{t,p} = \sum_{o \in \Phi^O} ge_{t,o,p} \left(fuelcost_{o,p} + OMcost_o \right) + st_{t,o,p} \cdot stcost_o \tag{34}$$

o^C is the oxidizations rate of thermal coal combustion, respectively, each equal to 99% and 83.7%(24). The parameter of CO_2 emissions per unit of standard coal fully burned is $\lambda^C = 2.76 \, tCO_2/tce$. The emission factor of natural gas generator is $e^G = 0.392 \, tCO_2/MWh(25)$ a_p^C is the provincial average standard coal consumption rate of coal-fired power plants in t/MWh, with the emissions factor provided in Supplementary Table 9. $CP_{t,p}$ presents the hourly cost by generator in province p.

However, not all provinces exclusively rely on locally generated electricity. Interprovincial transmission enables electricity consumption across regions while attributing emissions locally. To account for the emission disparity arising from power transmission, we derive CO_2 emissions from the consumer side using Eq. (35) and (36). Eq. (35) shows the proportion of power transmitted from province p to q concerning power generation. CO_2 emissions from the consumer side are computed by summing CO_2 emissions from power transmission and CO_2 emissions from locally consumed electricity.

$$TRcoff_{t,p,q} = \frac{tr_{t,p,q}}{\sum_{o \in \Phi^O} ge_{t,o,p}} \tag{35}$$

$$EC_p^{Total} = \sum_{t \in \Phi^T} \left[\left(1 - TRcoff_{t,p,q}\right) \cdot EP_{t,p} + \sum_{q \in \Phi^P} TRcoff_{t,q,p} \cdot EP_{t,q} \right] \tag{36}$$

Correspondingly, system cost from the consumer side is shown in Eq. (34):

$$CC_p^{Total} = \sum_{t \in \Phi^T} \left[\left(1 - TRcoff_{t,p,q}\right) \cdot CP_{t,p} + \sum_{q \in \Phi^P} TRcoff_{t,q,p} \cdot CP_{t,q} \right] \tag{37}$$

Supplementary Fig. 4 illustrates the CO_2 intensity and per-unit electricity cost for each province's electricity consumption. CO_2 intensity is determined by dividing a province's total CO_2 emissions by its electricity consumption, while electricity cost is calculated by dividing the total power system cost by electricity consumption. Across different weather conditions, the lowest CO_2 intensity and

electricity costs are observed in EWYs, with the highest values appearing in ECYs. Except for Sichuan, all provinces can reduce their CO_2 intensity and electricity costs through RAC replacement. However, in Sichuan, both CO_2 intensity and electricity cost slightly increase with RAC replacement. Among the provinces, Sichuan and Yunnan have the lowest emissions and costs per kilowatt-hour. As the loads in these provinces decrease due to RAC replacement, those with higher generation costs can primarily meet their needs by purchasing lower-cost electricity from provinces like Sichuan.

Supplementary Table 1. China's Energy-efficiency Label Grade Thresholds for RACs

Issuance Year	File Number	Type and Performance Index	Cooling Capacity	Grade 5	Grade 4	Grade 3	Grade 2	Grade 1	Substituted File
2000	GB12021.3-2000	Fixed Speed (EER)	≤2500W			2.5			
		Fixed Speed (EER)	2500W-4500W			2.45			
2004	GB12021.3-2004	Fixed Speed (EER)	≤4500W	2.6	2.8	3	3.2	3.4	GB12021.3-2000
2008	GB21455-2008	Cooling-only and Heat Pump Inverter (SEER)	≤4500W	3	3.4	3.9	4.5	5.2	
2010	GB12021.3-2010	Fixed Speed (EER)	≤4500W			3.2	3.4	3.6	GB12021.3-2004
2013	GB21455-2013	Cooling-only Inverter (SEER)	≤4500W			4.3	5	5.4	GB21455-2008
		Heat Pumps Inverter (APF)	≤4500W			3.5	4	4.5	
2019	GB21455-2019	Fixed Speed and Cooling-only Inverter (SEER)	≤4500W	3.7	3.9	5	5.4	5.8	GB21455-2013, GB12021.3-2010
		Heat Pumps Inverter (APF)	≤4500W	3.3	3.5	4	4.5	5	

Supplementary Table 2. Abbreviation and affiliation of provinces in southern China

Province	Abbreviation of province name	Region Grid	Climate Zone	Coastal or Inland
Shanghai	SH	East China Power Grid	Hot Summer Cold Winter	Coastal
Jiangsu	JS	East China Power Grid	Hot Summer Cold Winter	Coastal
Zhejiang	ZJ	East China Power Grid	Hot Summer Cold Winter	Coastal
Anhui	AH	East China Power Grid	Hot Summer Cold Winter	Inland
Fujian	FJ	East China Power Grid	Hot Summer Warm Winter	Coastal
Jiangxi	JX	Central China Power Grid	Hot Summer Cold Winter	Inland
Hubei	HB	Central China Power Grid	Hot Summer Cold Winter	Inland
Hunan	HN	Central China Power Grid	Hot Summer Cold Winter	Inland
Guangdong	GD	Southern China Power Grid	Hot Summer Warm Winter	Coastal
Guangxi	GX	Southern China Power Grid	Hot Summer Warm Winter	Coastal
Hainan	HA	Southern China Power Grid	Hot Summer Warm Winter	Coastal
Chongqing	CQ	Central China Power Grid	Hot Summer Cold Winter	Inland
Sichuan	SC	Central China Power Grid	Hot Summer Cold Winter	Inland
Guizhou	GZ	Southern China Power Grid	Temperate	Inland
Yunnan	YN	Southern China Power Grid	Temperate	Inland

Supplementary Table 3. Parameter Settings of Power Generation(11, 15) (MW)

Technology	Maximum Capacity Factor	Ramp up and Ramp down Constraint	Fuel Cost(yuan/MWh)	Variable O&M Cost(yuan/MWh)	Start-up Cost(yuan/MWh)
Coal	0.95	0.2	As shown in Table 9	29.9	1100
Gas	0.95	1	447	19.9	300
Biomass	1	1	435	71	50
Wind	1	1	0	8	0
Solar	1	1	0	39.8	0
Hydro	1	1	0	9.3	0
Pumpstorage	1	1	0	9.3	0

Supplementary Table 4. Annual Utilization Hours of Wind and Solar Power in Typical Weather Years(14)

Province	Wind	Solar
Shanghai	2289	861
Jiangsu	2001	1165
Zhejiang	2131	998
Anhui	189	1071
Fujian	2880	1063
Jiangxi	2104	917
Hubei	1881	1013
Hunan	2028	902
Guangdong	2096	1020
Guangxi	2744	1085
Hainan	1984	1061
Chongqing	2149	655
Sichuan	2537	1466
Guizhou	2049	1032
Yunnan	2857	1332

Supplementary Table 5. Installed Capacity of Lithium-ion Battery Storage in AE-S scenario(20) (MW)

Province	Lithium-ion Battery Storage
Shanghai	11517
Jiangsu	42770
Zhejiang	29592
Anhui	23312
Fujian	21435
Jiangxi	14751
Hubei	23947
Hunan	23297
Guangdong	59896
Guangxi	16707
Hainan	1800
Chongqing	16529
Sichuan	28986
Guizhou	2214
Yunnan	227

Supplementary Table 6. Parameters of 4-hour Lithium-ion Battery Systems(20, 26)

**Battery Cost Projections (yuan/MW)**	2320505
**Round-trip Efficiency**	85%
**Depth of Discharge**	25%
**Full Charge Time**	5 hours

Supplementary Table 7. Installed Capacity of Different Technologies in 2020(14) (MW)

Province	Coal	Gas	Biomass	Wind	Solar	Hydro	Pstorage	Nuclear
Shanghai	15020	7660	430	820	1370	0	0	0
Jiangsu	79210	17000	2420	15470	16840	368	2600	5490
Zhejiang	47380	12620	2400	1860	15170	2333	4580	9110
Anhui	51430	140	2130	4120	13700	905	3180	0
Fujian	28620	3910	800	4860	2020	3337	1200	8710
Jiangxi	20560	140	800	5100	7760	1578	1200	0
Hubei	28910	1120	1100	5020	6980	19278	1270	0
Hunan	20240	80	840	6690	3910	6775	1200	0
Guangdong	66200	26960	2830	5650	7970	2813	7280	16140
Guangxi	18590	540	2070	6530	2050	7458	0	2170
Hainan	3240	1690	460	290	1430	168	600	1300
Chongqing	13290	1490	360	970	670	3317	0	0
Sichuan	12120	1280	840	4260	1910	41181	0	0
Guizhou	34690	560	350	5800	10570	9455	0	0
Yunnan	12980	30	492	8810	3880	36042	0	0

Note: Nuclear energy generation has been adjusted by multiplying it with its capacity factor and is represented as non-dispatched in our analysis. Pstorage denotes the pumped storage.

Supplementary Table 8. Changed Installed Capacity under 2WS and 4WS Capacities Case (MW)

Capacity Case		2WS			4WS	
**Province**	**Coal**	**Wind**	**Solar**	**Coal**	**Wind**	**Solar**
Shanghai	14671	1640	2740	13624	3280	5480
Jiangsu	73437	30940	33680	56117	61880	67360
Zhejiang	45199	3720	30340	38657	7440	60680
Anhui	49666	8240	27400	44375	16480	54800
Fujian	26777	9720	4040	21248	19440	8080
Jiangxi	18523	10200	15520	12411	20400	31040
Hubei	27025	10040	13960	21370	20080	27920
Hunan	18289	13380	7820	12434	26760	15640
Guangdong	63920	11300	15940	57080	22600	31880
Guangxi	16291	13060	4100	9392	26120	8200
Hainan	3001	580	2860	2284	1160	5720
Chongqing	13002	1940	1340	12138	3880	2680
Sichuan	10567	8520	3820	5906	17040	7640
Guizhou	32088	11600	21140	24282	23200	42280
Yunnan	9517	17620	7760	0	35240	15520

Supplementary Table 9. Coal-fired Power Plants Emissions Factor and Fuel Cost(14) (MWh)

**Province**	**Standard Coal Consumption Rate (kg)**	**Emission Factor (tCO_2)**	**Fuel Cost (yuan)**
Shanghai	293.1	0.80	249.9
Jiangsu	290.2	0.79	247.5
Zhejiang	296.1	0.81	252.5
Anhui	297.8	0.81	253.9
Fujian	305.6	0.84	260.6
Jiangxi	300.7	0.82	256.4
Hubei	298.6	0.82	254.6
Hunan	308.4	0.84	263.0
Guangdong	294.8	0.81	251.4
Guangxi	311.3	0.85	265.5
Hainan	302	0.83	257.5
Chongqing	311.6	0.85	265.7
Sichuan	325	0.89	277.1
Guizhou	323.4	0.88	275.8
Yunnan	338.5	0.92	288.6
National Average	302.6	0.83	258.0

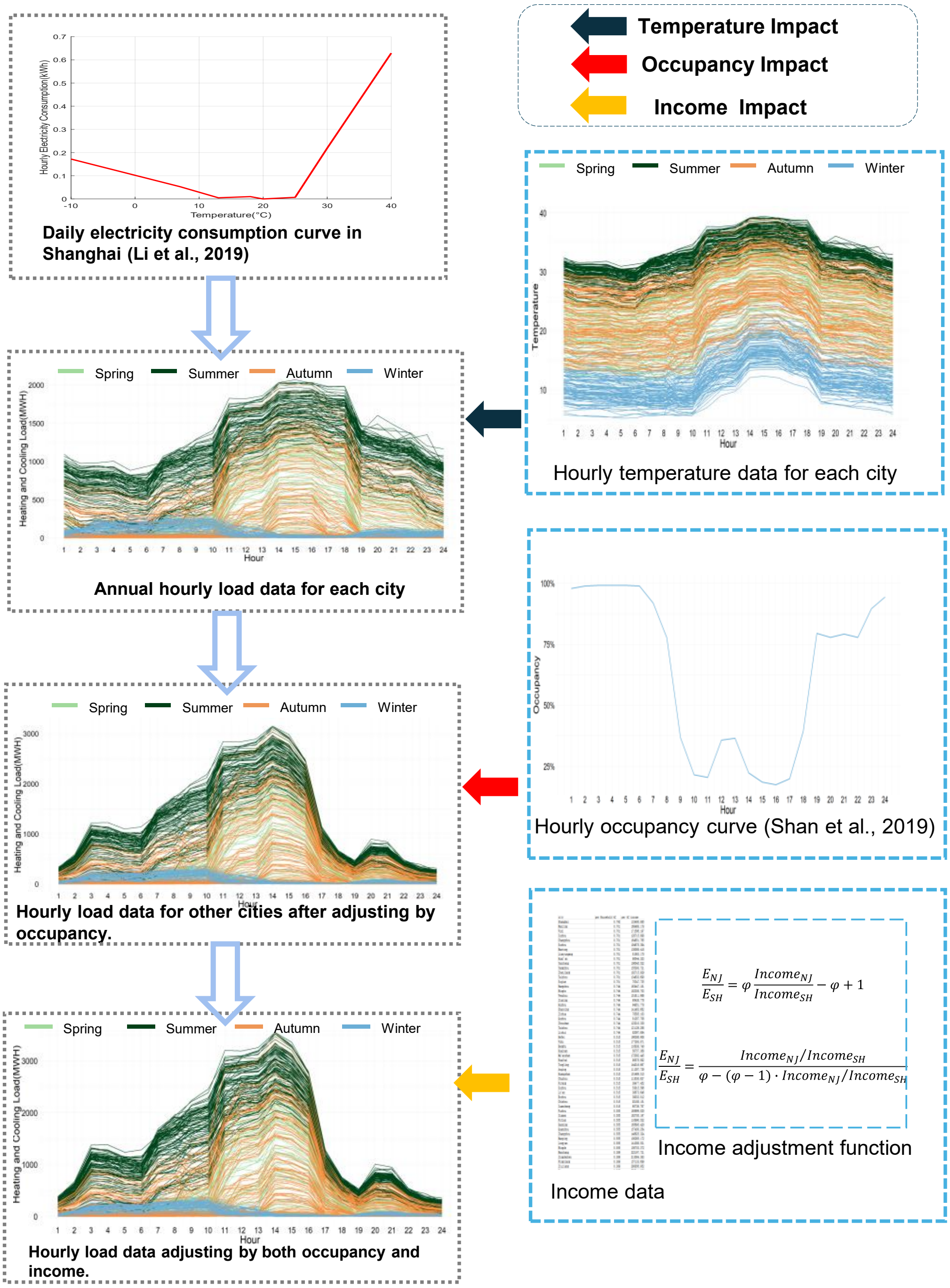

Supplementary Figure 1. Technical Procedure for heating and cooling load calculations

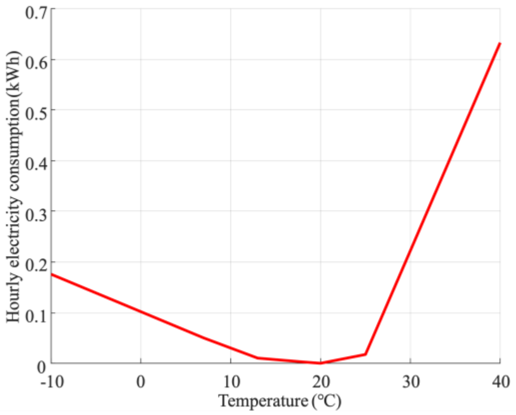

Supplementary Figure 2. The U-shaped piecewise function of heating and cooling load in Shanghai

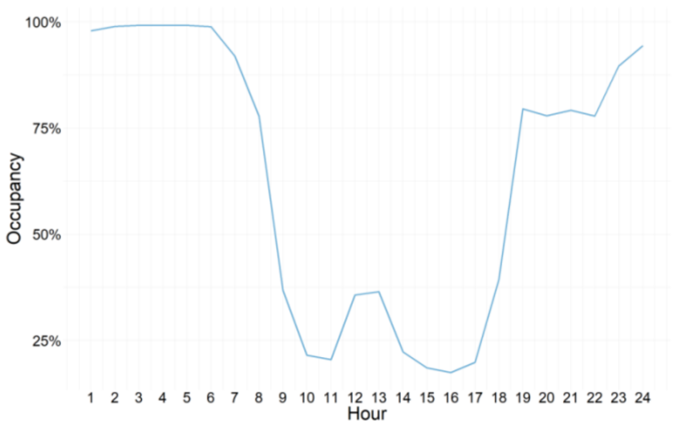

Supplementary Figure 3. The Hourly Occupancy Rates of a Typical Day in Chengdu

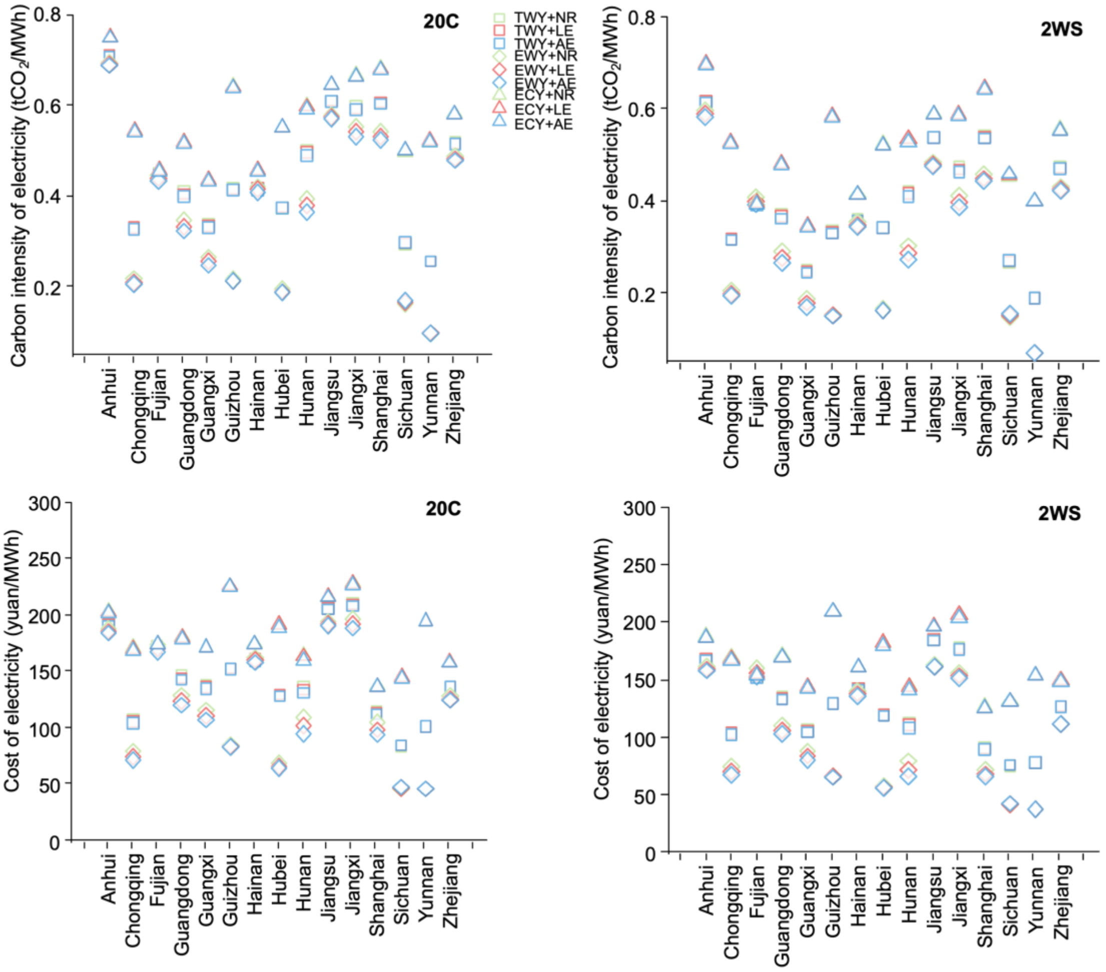

Supplementary Figure 4. CO_2 intensity and electricity cost of provinces in southern China

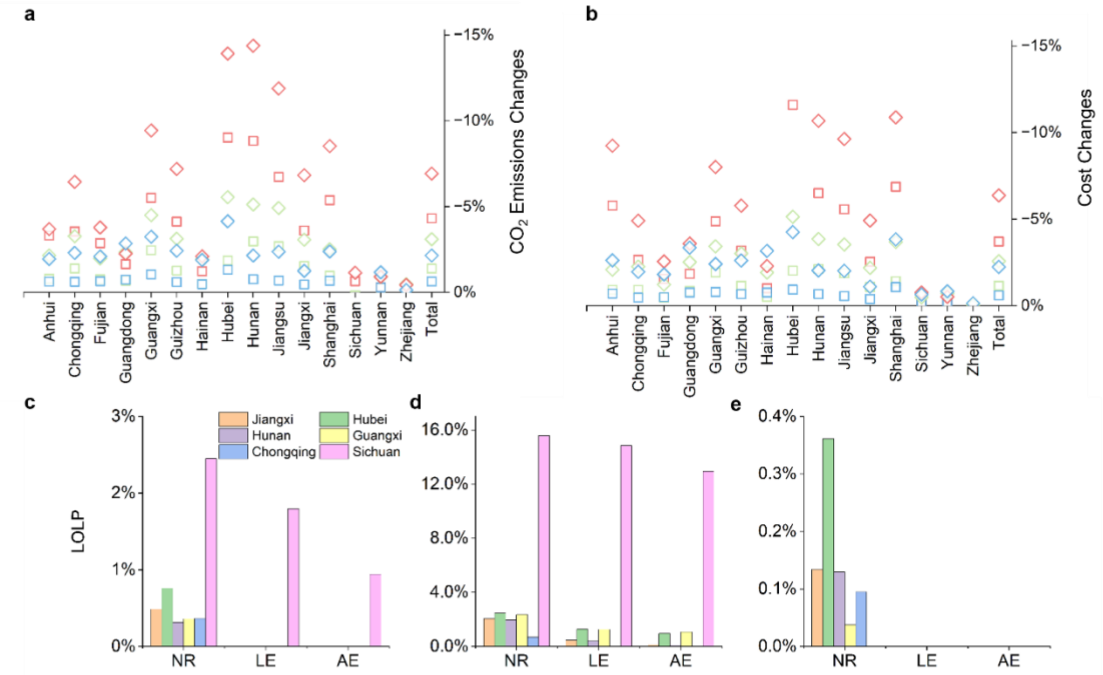

Supplementary Figure 5. CO_2 emissions reduction(a), cost-saving(b) and LOLP changes of provinces under TWYs(c), ECYs(d), and EWYs(e) scenarios in southern China under 4WS case

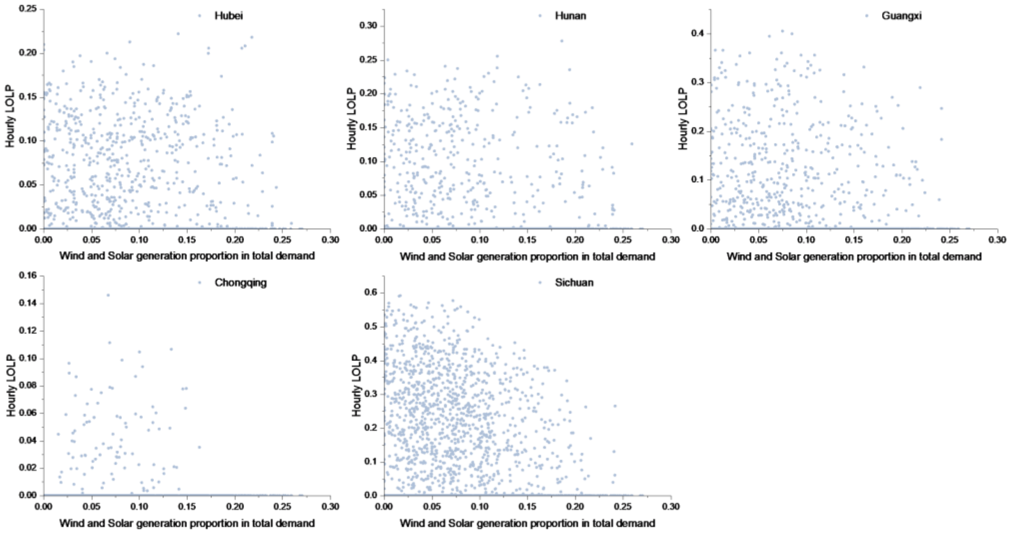

Supplementary Figure 6. Hourly LOLP variation with the proportion of wind and solar generation in meeting hourly total power demand for Hubei, Hunan, Guangxi, Chongqing, and Sichuan under the 20C capacity case and ECY weather conditions in the NR replacement scenario.

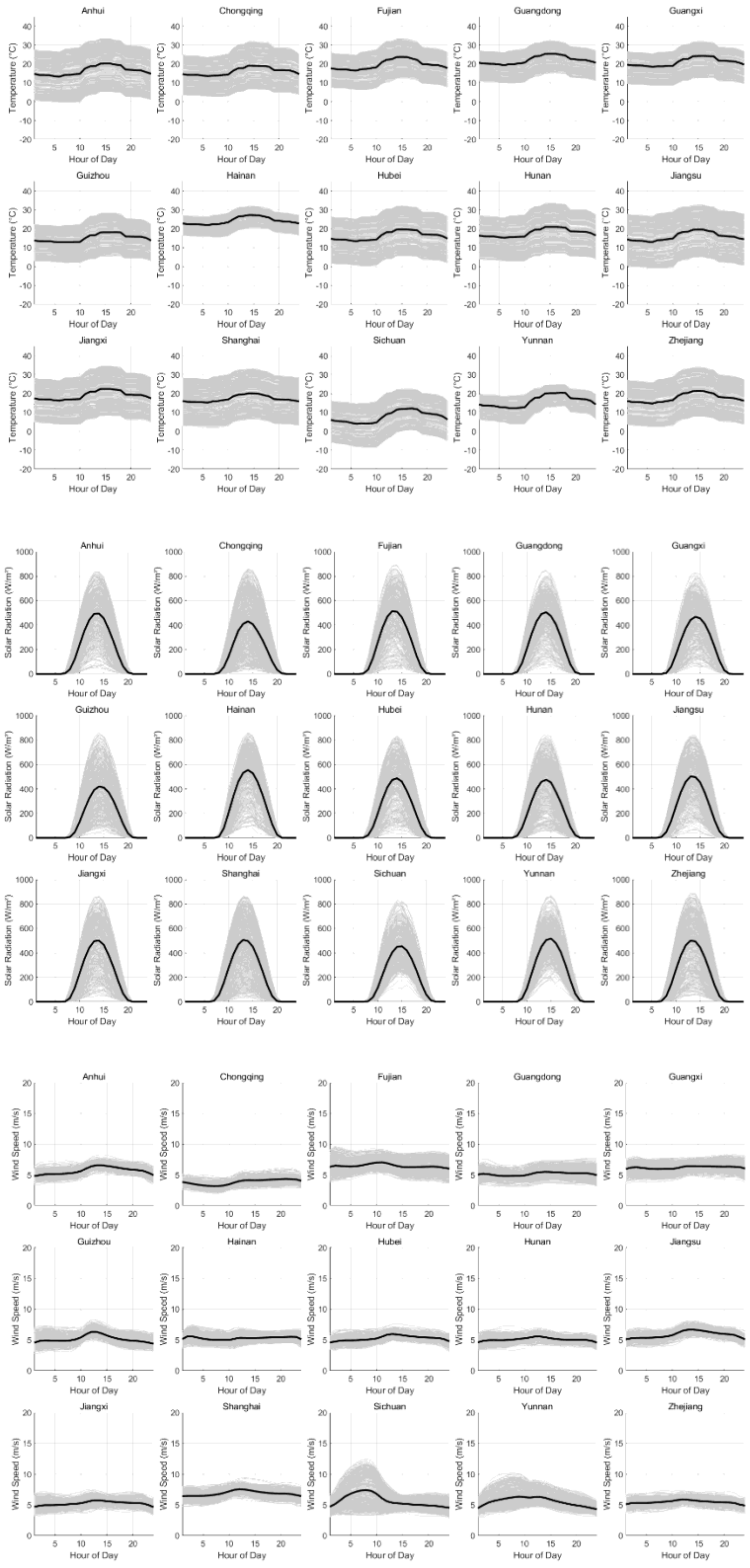

Supplementary Figure 7. Daily weather patterns (temperature, solar radiation, and wind speed) in TWYs. The black line shows the average value of 365 days, while the grey lines show the weather data for each individual day.

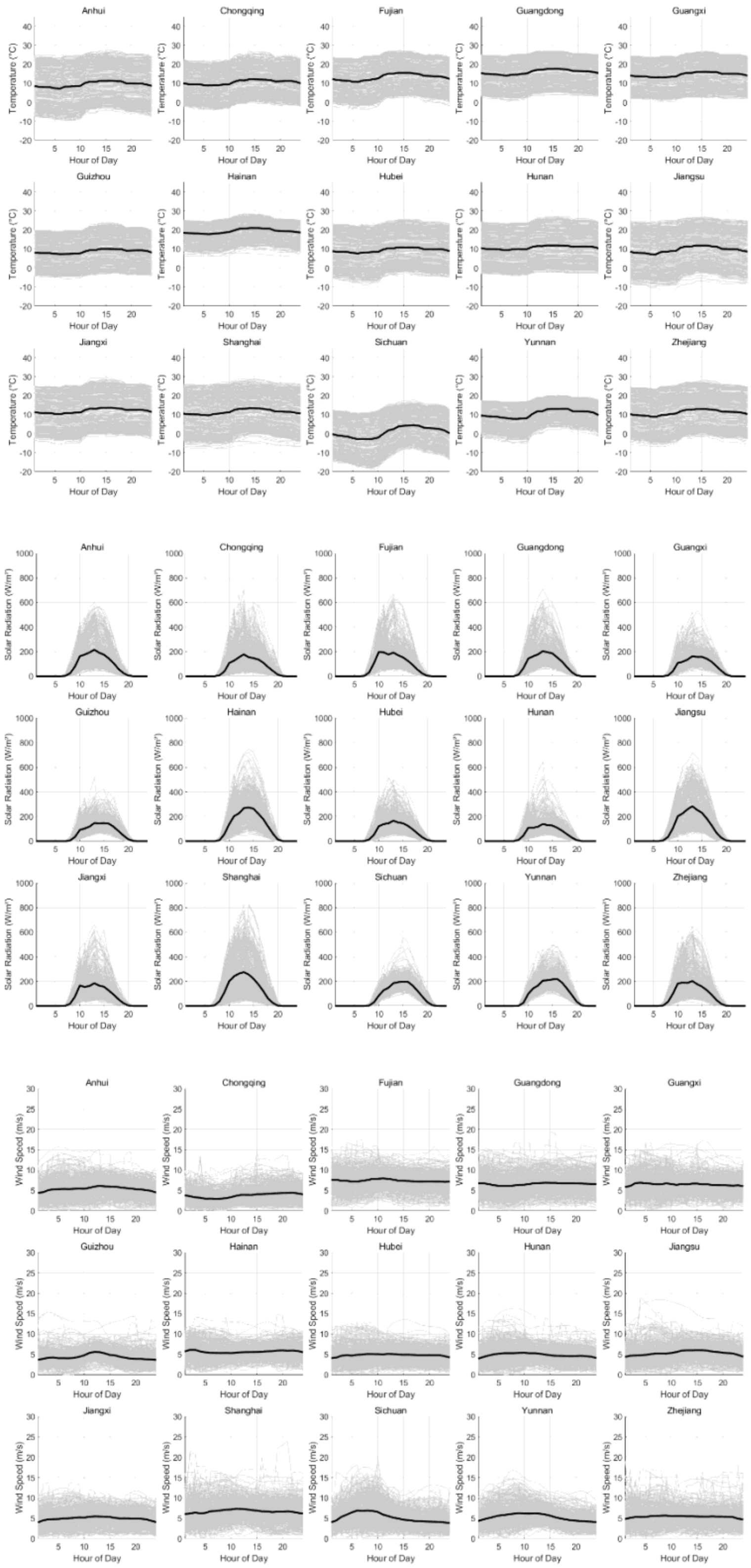

Supplementary Figure 8. Daily weather patterns (temperature, solar radiation, and wind speed) in ECYs. The black line shows the average value of 365 days, while the grey lines show the weather data for each individual day.

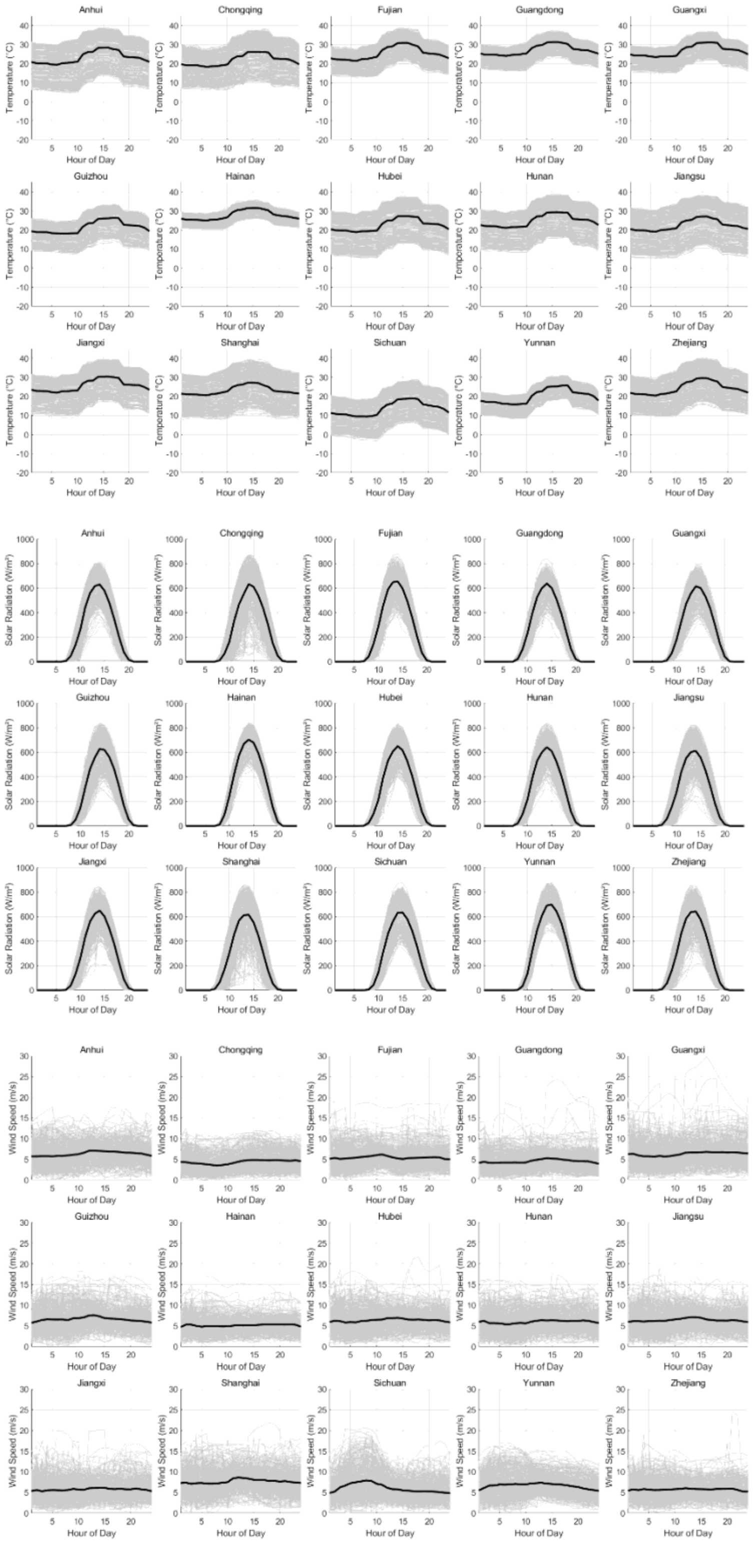

Supplementary Figure 9. Daily weather patterns (temperature, solar radiation, and wind speed) in EWYs. The black line shows the average value of 365 days, while the grey lines show the weather data for each individual day.

Supplementary References

1. National Development and Reform Commission (NDRC), The Advanced Level, Energy Saving Level and Access Level of Energy Efficiency of Key Energy-Using Products and Equipment. (2022).
2. Wu G, Ren T, Ding G, Cheng J, A method to evaluate APF for room air conditioner with on/off compressor by using rated performance data. *J. Refrig.* 88–93 (2016). https://doi.org/10.3969/j.issn.0253-4339.2016.03.088.
3. N. Karali, *et al.*, Improving the energy efficiency of room air conditioners in China: Costs and benefits. *Appl. Energy* **258**, 114023 (2020).
4. V. Lebakula, *et al.*, LandScan Silver Edition. Oak Ridge National Laboratory. https://doi.org/10.48690/1531770. Deposited 2024.
5. National Bureau of Statistics of China, "Communiqué of the Seventh National Population Census (No. 7)" (2021).
6. X. Wang, C. Ding, W. Cai, L. Luo, M. Chen, Identifying household cooling savings potential in the hot summer and cold winter climate zone in China: A stochastic demand frontier approach. *Energy* **237**, 121588 (2021).
7. Y. Li, W. A. Pizer, L. Wu, Climate change and residential electricity consumption in the Yangtze River Delta, China. *Proc. Natl. Acad. Sci.* **116**, 472–477 (2019).
8. H. Jiang, *et al.*, How do urban residents use energy for winter heating at home? A large-scale survey in the hot summer and cold winter climate zone in the Yangtze River region. *Energy Build.* **223**, 110131 (2020).
9. M. Zhou, *et al.*, Environmental benefits and household costs of clean heating options in northern China. *Nat. Sustain.* **5**, 329–338 (2021).
10. M. Luo, B. Cao, J. Damiens, B. Lin, Y. Zhu, Evaluating thermal comfort in mixed-mode buildings: A field study in a subtropical climate. *Build. Environ.* **88**, 46–54 (2015).
11. Z. Zhuo, *et al.*, Cost increase in the electricity supply to achieve carbon neutrality in China. *Nat. Commun.* **13**, 3172 (2022).
12. K. Du, Y. Yu, C. Wei, Climatic impact on China's residential electricity consumption: Does the income level matter? *China Econ. Rev.* **63**, 101520 (2020).
13. S. Hu, D. Yan, J. An, S. Guo, M. Qian, Investigation and analysis of Chinese residential building occupancy with large-scale questionnaire surveys. *Energy Build.* **193**, 289–304 (2019).
14. China Electricity Council, "China Electricity Industry Economic Operation Report 2020" (2021).
15. X. Yao, B. Yi, Y. Yu, Y. Fan, L. Zhu, Economic analysis of grid integration of variable solar and wind power with conventional power system. *Appl. Energy* **264**, 114706 (2020).
16. L. Rudolf, *et al.*, Influence of Temperature and Transmitted Power on Losses in Particular Transmission System. *WSEAS Trans. POWER Syst.* **17**, 53–61 (2022).
17. A. R. Jordehi, How to deal with uncertainties in electric power systems? A review. *Renew. Sustain. Energy Rev.* **96**, 145–155 (2018).
18. W. Hu, Z. Liu, J. Tan, "Thermodynamic Analysis of Wind Energy Systems" in *Wind Solar Hybrid Renewable Energy System*, K. E. Okedu, A. Tahour, A. G. Aissaou, Eds. (IntechOpen, 2019).
19. A. L. Bukar, C. W. Tan, K. Y. Lau, Optimal sizing of an autonomous

photovoltaic/wind/battery/diesel generator microgrid using grasshopper optimization algorithm. *Sol. Energy* **188**, 685–696 (2019).

20. W. Cole, A. Frazier, C. Augustine, "Cost Projections for Utility-Scale Battery Storage: 2021 Update" (2021).
21. X. Yao, Y. Fan, F. Zhao, S.-C. Ma, Economic and climate benefits of vehicle-to-grid for low-carbon transitions of power systems: A case study of China's 2030 renewable energy target. *J. Clean. Prod.* **330**, 129833 (2022).
22. J.-L. Fan, *et al.*, Impacts of climate change on hydropower generation in China. *Math. Comput. Simul.* **167**, 4–18 (2020).
23. Sorenson B, "Renewable Power Generation Costs in 2019" (IRENA, 2020).
24. Z. Liu, *et al.*, Reduced carbon emission estimates from fossil fuel combustion and cement production in China. *Nature* **524**, 335–338 (2015).
25. MEE, "2019-2020 Implementation Plan for National Carbon Emission Trading Total Allowances Setting and Allocation" (2020).
26. B. Velev, B. Djudzhev, V. Dimitrov, N. Hinov, Comparative Analysis of Lithium-Ion Batteries for Urban Electric/Hybrid Electric Vehicles. *Batteries* **10**, 186 (2024).
